# Web-Based and Mobile Stress Management Intervention for Employees: A Randomized Controlled Trial

**DOI:** 10.2196/jmir.5112

**Published:** 2016-01-27

**Authors:** Elena Heber, Dirk Lehr, David Daniel Ebert, Matthias Berking, Heleen Riper

**Affiliations:** ^1^ Leuphana University Lueneburg Division of Online Health Training, Innovation Incubator Lueneburg Germany; ^2^ Leuphana University Lueneburg Institute of Psychology Lueneburg Germany; ^3^ Friedrich-Alexander-University Erlangen-Nuremberg Department of Clinical Psychology and Psychotherapy Erlangen Germany; ^4^ VU University Amsterdam Department of Clinical Psychology Amsterdam Netherlands; ^5^ Telepsychiatric Centre University of Southern Denmark Odense Denmark

**Keywords:** Internet, randomized controlled trial, work, stress, stress management, mental health

## Abstract

**Background:**

Work-related stress is highly prevalent among employees and is associated with adverse mental health consequences. Web-based interventions offer the opportunity to deliver effective solutions on a large scale; however, the evidence is limited and the results conflicting.

**Objective:**

This randomized controlled trial evaluated the efficacy of guided Web- and mobile-based stress management training for employees.

**Methods:**

A total of 264 employees with elevated symptoms of stress (Perceived Stress Scale-10, PSS-10≥22) were recruited from the general working population and randomly assigned to an Internet-based stress management intervention (iSMI) or waitlist control group. The intervention (GET.ON Stress) was based on Lazarus’s transactional model of stress, consisted of seven sessions, and applied both well-established problem solving and more recently developed emotion regulation strategies. Participants also had the opportunity to request automatic text messages on their mobile phone along with the iSMI. Participants received written feedback on every completed session from an e-coach. The primary outcome was perceived stress (PSS-10). Web-based self-report assessments for both groups were scheduled at baseline, 7 weeks, and 6 months. At 12 months, an extended follow-up was carried out for the iSMI group only.

**Results:**

An intention-to-treat analysis of covariance revealed significantly large effect differences between iSMI and waitlist control groups for perceived stress at posttest (*F*_1,261_=58.08, *P*<.001; Cohen’s *d*=0.83) and at the 6-month follow-up (*F*_1,261_=80.17, *P*<.001; Cohen’s *d*=1.02). The effects in the iSMI group were maintained at 12-month follow-up.

**Conclusions:**

This Web- and mobile-based intervention has proven effective in reducing stress in employees in the long term. Internet-based stress management interventions should be further pursued as a valuable alternative to face-to-face interventions.

**Trial Registration:**

German Clinical Trials Register (DRKS): 00004749; http://drks-neu.uniklinik-freiburg.de/ drks_web/setLocale_EN.do (Archived by WebCite at http://www.webcitation.org/6e8rl98nl)

##  Introduction

Stress and related adverse outcomes for physical and mental health are highly prevalent and pose a major threat to public health. Individuals with high stress levels face various negative consequences of stress including sleeping problems [1], burnout [2], an increased risk of depression, anxiety [3], and coronary heart disease [4,5]. According to a recent survey [6], 31% of US employees feel tense or stressed on a daily basis. Meanwhile, 64% report receiving insufficient stress management resources from their employers.

In recent years, Web-based and mobile-based interventions for coping with work-related stress have emerged. The advantages attributed to Web-based interventions include the potential for large-scale delivery, 24/7 availability, low costs and a low-access threshold [7]. Moreover, a recent meta-analysis reveals an equivalence between face-to-face and Internet-based guided cognitive behavioral therapy [8]. For populations experiencing high levels of work-related stress, Web-based interventions can be an appealing method for flexibly integrating stress management exercises into daily life. In particular, mobile behavioral intervention technologies for mental health offer the potential to deliver training components in real time and the real world [9]. Internet-based interventions may also reach those who are unwilling to participate in traditional face-to-face interventions [10].

Face-to-face training on stress management has been proven to be effective [11-13]. However, the evidence base for Internet-based stress management interventions (iSMIs) remains inconclusive, as only a limited number of randomized controlled trials (RCTs) have been conducted. Some of these studies showed a significant moderate reduction of stress for Web-based interventions compared with a waitlist group [14-17], a no-treatment group [18], and an attention control group [19]. Other studies did not find beneficial between-group effects for stress at posttest [20-22]. For instance, Wolever et al [18] found an effect size of *d*=0.74 for reduced stress for a guided mindfulness at work intervention, whereas Wiegand et al [21] did not find significant between-group effects for an unguided comprehensive Web program that included olfactory care products for women. This lack of conclusiveness of iSMIs also applies to other mental health indicators, such as depression. The differences in effectiveness may result from variations in the type and length of interventions studied, the usage of guidance, the outcomes, the measurements, or the setting. Likewise, little is known about the long-term efficacy of iSMIs. Two RCTs have investigated the efficacy of iSMIs at the 6-month follow-up relative to a control group finding a non-significant effect for stress reduction in students [23] and a small to moderate effect for the general population [14]. An extended follow-up conducted by Ruwaard et al [16] over a 3-year period yielded beneficial results for reducing stress. However, no RCTs investigating an intervention combining Web-based and mobile components with a focus on stress reduction have addressed employees as a target group relative to a control group in long-term follow-ups (eg, 6 months). With regard to the content of such interventions, currently available iSMIs do not base their theoretical foundation on a specific stress model such as the effort-reward imbalance model [24] or the job-demand control model [25]. Likewise, more generic, established models of stress, such as Lazarus’s transactional model of stress [26] are not applied. Lazarus’s transactional model of stress specifies two coping strategies. Problem-focused coping is used to actively influence a stress situation in a positive way through the use of cognitive or behavioral efforts. Emotion-focused coping primarily serves the function of managing difficult emotions such as anger, disappointment, and sadness in relation to the specific situation. On the one hand, employees are often faced with problems that theoretically can be solved. Problem solving [27] is an established therapeutic method in dealing with such problems and has been proven to be successful in reducing mental and physical health problems [28]. This method has also been effectively used in Web-based interventions to manage depression, anxiety, and stress [29], although mixed results have been observed in studies targeting employees with depressive symptoms [30,31]. On the other hand, the working context also frequently requires dealing with problems that are unsolvable; such situations are commonly accompanied by strong negative emotions and require effective strategies on how to regulate these emotions. Emotion regulation skills have been shown to be relevant and successful in a broad range of mental disorders including depression and anxiety [32]; nevertheless, they remain largely untargeted in research on stress management interventions. From a theoretical perspective, promoting problem- and emotion-focused coping skills according to Lazarus’s model as two major intervention components within the same intervention appears promising; however, this approach has not yet been introduced. This study aimed to fill this gap in the research by investigating an iSMI based on the combination of problem solving and emotion regulation.

This paper presents the results of a waitlist-controlled randomized trial to investigate the efficacy of a newly developed iSMI that includes mobile components for reducing stress in employees with elevated stress levels. We assessed whether the participants in the intervention group (iSMI) reported significantly lower scores on the primary outcome of perceived stress on the Perceived Stress Scale-10 (PSS-10) at posttest and at 6-month follow-up as compared to those in a waitlist control (WLC) group. Among the secondary outcomes, selected mental- and work-related health indicators often perceived to arise due to chronic stress, such as depression, anxiety, and emotional exhaustion, were also considered.

## Methods

### Trial Design

Using a 2-arm randomized controlled design, 264 participants were randomly allocated (at a ratio of 1:1 and a block size of 2) to the iSMI or to a WLC group. Both groups had full access to treatment as usual.

### Participants

Participants 18 years and older were included if they were currently employed and scored 22 or above on the PSS-10. Due to the fact that the PSS is not a diagnostic measurement and there is no official cut-off available, we decided to use one standard deviation (SD 6.2) above the mean (PSS-10=15.3) in a large working population [33] as a cut-off value to choose participants with an elevated level of stress. We excluded any applicants who were at risk of suicide (Beck Suicide Item >1; [34]) or self-reported to have been previously diagnosed with dissociative or psychotic symptoms. Participants were recruited in Germany from January to October 2013 in the general working population through newspaper articles and announcements by the ministry of education. Primarily, they were recruited through a large German health insurance company. The intervention addressed employees who were frequently stressed or exhausted, who felt that problems were increasingly difficult to handle, and who struggled to cope with difficult emotions. The intervention was advertised in the print membership magazine of a large German health insurance company. It was stated that, in cooperation with a university, the health insurance company offered this online training to employees, provided they were suitable for study inclusion. Moreover, the advantages of the intervention such as 24/7 availability, personal e-coaching, and participation free of charge were delineated. Those interested in participating had to provide an email address and a first and last name that could be pseudonyms if desired. Individuals received a link to the online screening questionnaire via email. Provided they were eligible, applicants had to submit their signed informed consent via regular post or scanned via email. Upon receipt of the informed consent, participants had to complete all baseline questionnaires. Subsequently, they were randomized into either the intervention or the waitlist control group.

### Intervention

The iSMI GET.ON Stress is based on Lazarus’s transactional model of stress [26]. This intervention applied both well-established problem solving and more recently developed emotion regulation strategies. Important principles for health behavior change such as goal setting, action planning, and coping planning were followed. The iSMI consisted of seven sessions and a booster session provided 4 weeks after training completion. Following psycho-education (Session 1), the participants learned a 6-step procedure to systematically solve problems (Sessions 2-3). In Sessions 4-6, the participants were introduced to emotion regulation techniques (muscle- and breathing relaxation, acceptance of negative emotions, and self-support in difficult situations). Session 7 included a plan for the future. The iSMI was specifically tailored to employees; this was reflected in the wording of the intervention, the example characters provided throughout the training, as well as in optional short informational material related to typical stress-related topics (eg, psychological detachment from work, time management, sleep hygiene, worrying, and organization of breaks during work) provided alongside the intervention. The application of exercises was strongly recommended. The participants were advised to complete 1-2 sessions per week. The program included interactive exercises, audio/video files, and downloadable material and was presented on a secured Web-based platform. Upon activation of the account through the research team, participants used their email address and a self-chosen password to log on. Within 48 hours after session completion, an e-coach provided approximately three quarters of a page of written, non-therapeutic feedback intended to increase adherence and motivation. The e-coaches reported that the average time spent per feedback was 30 minutes. In the event of non-completion of a session, they also sent reminders. Each e-coach had a degree in psychology and followed a standardized manual on feedback writing. Fidelity and adherence to the feedback manual was ensured by providing extensive coaching on feedback writing and by employing a psychotherapist who provided supervision for the e-coaches. The participants could receive automatic text messages on their mobile phone along with the iSMI (eg, short relaxation exercises: “Relax your muscles in your hands and arms for 3 seconds now. Follow your breathing and each time you breathe out, relax a little more.”) and were given the choice of either light (1 text message every other day) or intensive support (2-3 text messages per day) according to personal preferences. The text message coach was part of the intervention, aimed at reminding participants to practice and increasing the adherence to the intervention [9]. A more detailed description of the iSMI can be found in the protocol of the trial [35]. Screenshots of the intervention are available in the Multimedia Appendix 1.

### Measurements

All questionnaires were self-assessed online at baseline (T1), 7 weeks (T2, post-treatment), 6 months (T3), and 12 months (T4, iSMI group only) after randomization. The WLC group received access to the intervention following T3.

### Primary Outcome Measure

The primary outcome was the level of perceived stress as measured by the PSS-10 [36]. As this scale is based on Lazarus’s transactional model of stress, it fits well with the theoretical basis of the intervention. We further decided to employ a general stress scale as previous research in a similar intervention for employees showed that work-related and non-work-related problems are equally often indicated and addressed [30]. The items were answered using a 5-point Likert scale (0=never; 1=almost never; 2=sometimes; 3=fairly often; 4=very often; range 0-40) referring to the past week. Cronbach alphas for this scale have been reported to range from .78 to .91 [37] and was .70 at T1, .90 at T2, .90 at T3, and .91 at T4 in this study.

### Secondary Outcome Measures

#### Mental Health

Among the secondary outcomes concerning mental health, the following outcomes were measured using the specified scales: depression, using the Center for Epidemiological Studies’ Depression Scale (CES-D) [38] (20 items; range 0-60; α=.91); insomnia severity, using the Insomnia Severity Index (ISI) [39] (7 items; range 0-28; α=.90); anxiety, using the subscale of the Hospital Anxiety and Depression Scales (HADS-A) [40] (7 items; range 0-21; α=.83); worrying, using the Penn State Worry Questionnaire, Ultra Brief Version-past week (PSWQ-PW) [41] (3 items; range 0-18; α=.87); and quality of life, using the Short Form 12 (SF-12) PH (physical health) and MH (mental health) [42].

#### Work-Related Health

Within the area of work-related health, we assessed emotional exhaustion, using the subscale of the Maslach Burnout Inventory (MBI-EE) [43] (5 items; range 1-6; α=.89); work engagement, using the Utrecht Work Engagement Scale (UWES) [44] (9 items; range 0-6; α=.94); and psychological detachment, using the subscale of the Recovery Experience Questionnaire (REQ-PD) [45] (4 items; range 1-5; α=.93). Moreover, mean days of absenteeism and presenteeism within the previous 3 months were assessed using the respective items of the German Version of the Trimbos and Institute of Medical Technology Assessment Cost Questionnaire for Psychiatry (TiC-P-G) [46].

#### Skills/Competencies

Emotion regulation in terms of comprehension (-C), acceptance (-A), and self-support (-SS) of the Emotion Regulation Skills Questionnaire (ERSQ), using the ERSQ-27 [47] (9 items; range 0-4; α=.87, .86, .85), and general distress, using the Emotion Specific Version, ERSQ-ES-GD [48] (12 items; range 0-4; α=.88) were assessed as measures of skills/competencies.

#### Other Measures

Client satisfaction, using the Client Satisfaction Questionnaire (CSQ-8) [49], demographic variables, and reasons for dropout will also be reported.

### Sample Size

As the current evidence base for Web-based stress-management is limited and the effect sizes vary considerably between trials (from non-significant to moderate-large), we decided to use a conservative approach in order to also detect small effect sizes. We relied on a meta-analysis on traditional stress management interventions [13] and expected an effect size of *d*=0.35. Therefore, based on an alpha of .05 (two-tailed test), and a power of 80%, a sample size of 132 participants per group was necessary.

### Randomization

The applied random integer list was created by an independent researcher using a Web-based randomization program (Randlist). The participants were informed about the randomization outcome via email.

### Statistical Analyses

All analyses are reported according to the Consolidated Standards of Reporting Trials (CONSORT) statement regarding eHealth [50] using intention-to-treat (ITT) procedures (see Multimedia Appendix 2). Additionally, per-protocol and study completers-only analyses are reported. A significance level of .05 (two-sided) was used for all analyses. Analyses were performed using IBM SPSS version 22.

#### Missing Data

Multiple imputation was used to handle missing data [51]. Ten single imputations of the missing values were calculated based on the valid data for all outcome measures at all assessment points (T1, T2, T3, and T4) as well as age and gender and were aggregated into a single overall estimate of the effects of the intervention.

#### Intervention Effect

The iSMI and WLC groups were compared at 7 weeks (T2) and 6 months (T3) using analysis of covariance (ANCOVA) with baseline levels as covariates. Cohen’s *d* with 95% confidence intervals (CIs) was calculated based on the imputed dataset by comparing the means and SDs of the iSMI and WLC groups at the respective time points (eg, post-means and post-SDs). According to Cohen [52], *d*=0.2 can be considered a small effect, *d*=0.5 a medium effect, and *d*=0.8 a large effect.

#### Reliable Change

The clinical significance in terms of reliable change was calculated according to the method of Jacobson and Truax [53] using the following formula: 1.96 × SD1 × sqrt(2) × sqrt(1-rel). Thereby, we used the standard deviation of the norm population (SD 6.2) and the reliability of the PSS-10 scale (α=.91) according to Cohen’s and Janicki-Deverts’ samples in 2006 and 2009 [37]. The participants were defined as having reliably changed if their PSS-10 score differed more than (+/-) 5.16 points from T1-T2 and T1-T3.

#### Symptom-Free Status

According to Jacobson and Truax [53], a cut-off point indicating symptom-free status was calculated and defined as scoring more than 2 SDs below the mean (T1) of the stressed population.

#### Number Needed to Treat

The number needed to treat (NNT) indicates the number of participants who must be treated to generate one additional clinically significant change. NNTs and their 95% confidence intervals [54,55] were calculated for reliable change and symptom-free status.

All procedures involved in the study were consistent with the generally accepted standards of ethical practice and were approved by the ethical committee of the University of Marburg (reference number AZ 2012-43K).

## Results

### Participants

**Figure 1:**
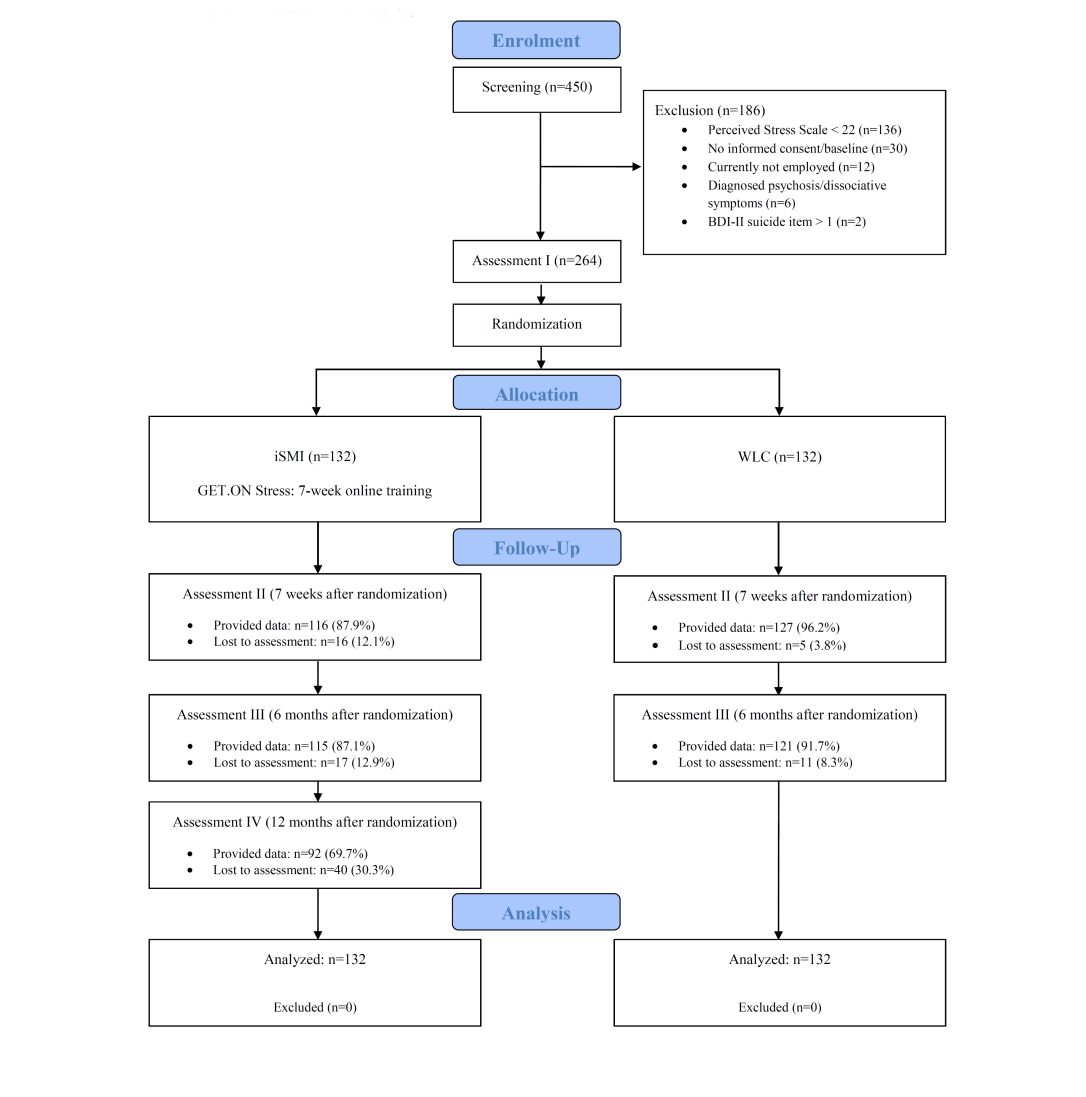
Flow of participants.

A total of 450 individuals were screened for eligibility, and 186 were excluded primarily because they scored below the cut-off (136/450), because of a lack of informed consent/baseline (30/450), or other reasons (20/450). The study flow is illustrated in Figure 1.

### Baseline Characteristics

The demographic variables for all study participants are displayed in Table 1.

The average age of the participants was 43.3 years (SD 10.2). The sample was primarily female (193/264, 73.1%), married or in a relationship (160/264, 60.6%), and highly educated (203/264, 76.9%). Most participants were employed full-time (204/264, 77.3%); their average working experience was 18.1 (SD 11.1) years; and they were working in various sectors, most frequently in the social sector (97/264, 36.7%). Only a small percentage of participants had previously taken part in any health training (34/264, 12.9%). Having received psychotherapy was indicated by 95 (36.0%) of the 264 participants and currently being in psychotherapy by 16/264 (6.1%). Table 2 summarizes all means and SDs for the iSMI and WLC groups.

**Table 1:** Baseline characteristics.

Characteristics		All participants (n=264)	iSMI (n=132)	WLC (n=132)
**Sociodemographic characteristics**				
	Age, mean (SD)	43.3 (10.2)	42.4 (10.7)	44.2 (9.6)
	Gender, female, n (%)	193 (73.1)	97 (73.5)	96 (72.7)
	Married or in a relationship, n (%)	160 (60.6)	80 (60.6)	80 (60.6)
**Ethnicity, n (%)**				
	Caucasian/white	220 (83.3)	110 (83.3)	110 (83.3)
	Prefer not to say	44 (16.7)	22 (16.7)	22 (16.7)
**Educational level, n (%)**				
	Low	5 (1.9)	3 (2.3)	2 (1.5)
	Middle	56 (21.2)	25 (18.9)	31 (23.5)
	High	203 (76.9)	104 (78.8)	99 (75.0)
**Work characteristics**				
	Full-time employed, n (%)	204 (77.3)	105 (79.5)	99 (75.0)
	Part-time employed, n (%)	57 (21.6)	25 (18.9)	32 (24.2)
	On sick leave, n (%)	3 (1.1)	2 (1.5)	1 (0.8)
	Work experience in years, mean (SD)	18.1 (11.1)	17.2 (10.8)	18.9 (11.2)
**Work sectors, n (%)**				
	Social	97 (36.7)	48 (36.4)	49 (37.1)
	Service	43 (16.3)	21 (15.9)	22 (16.7)
	Health	36 (13.6)	22 (16.7)	14 (10.6)
	Economy	31 (11.7)	14 (10.6)	17 (12.9)
	IT	15 (5.7)	8 (6.1)	7 (5.3)
	Others	42 (16.0)	19 (14.3)	23 (17.4)
**Income in Euro, per year, n (%)**				
	<10,000	10 (3.8)	7 (5.3)	3 (2.3)
	10,000-30,000	48 (18.2)	28 (21.2)	20 (15.2)
	30,000-40,000	63 (23.9)	26 (19.7)	37 (28.0)
	40,000-50,000	47 (17.8)	26 (19.7)	21 (15.9)
	50,000-60,000	29 (11.0)	15 (11.4)	14 (10.6)
	60,000-100,00	32 (12.1)	17 (12.9)	15 (11.4)
	>100,000	4 (1.5)	1 (0.8)	3 (2.3)
	Prefer not to say	31 (11.7)	12 (9.1)	19 (14.4)
**Experience, n (%)**				
	Previous health training	34 (12.9)	17 (12.9)	17 (12.9)
	Previous psychotherapy	95 (36.0)	52 (39.4)	43 (32.6)
	Current psychotherapy	16 (6.1)	5 (3.8)	11 (8.3)

**Table 2:** Means and standard deviations for the iSMI group and the WLC group (ITT sample).

		T1				T2^a^				T3^a^				T4^a,b^	
		iSMI		WLC		iSMI		WLC		iSMI		WLC		iSMI	
Outcome		M	SD	M	SD	M	SD	M	SD	M	SD	M	SD	M	SD
**Primary outcome**															
	PSS-10	25.89	3.85	25.15	3.96	17.88	6.17	22.96	6.07	16.08	6.03	22.10	5.81	16.25	6.35
**Mental health**															
	CES-D	23.34	8.47	23.77	7.59	15.61	9.09	21.35	8.82	13.83	7.71	21.49	8.48	14.56	8.71
	ISI	15.53	6.62	14.82	6.48	8.79	5.81	11.20	6.15	8.18	5.58	11.00	5.35	7.82	4.95
	HADS-A	11.23	3.34	10.67	3.36	7.79	3.98	10.32	3.46	6.73	3.38	9.65	3.58	6.51	3.46
	PSWQ-PW	10.17	3.98	10.23	3.74	6.82	3.99	8.91	4.21	6.08	3.78	8.65	4.36	5.83	3.82
	SF-12 MH^c^	32.29	8.44	32.55	8.08	N/A	N/A	N/A	N/A	43.38	10.56	36.54	9.50	44.16	9.69
	SF-12 PH^c^	48.24	10.05	48.44	9.43	N/A	N/A	N/A	N/A	49.23	8.17	47.57	8.75	49.27	6.39
**Work-related health**															
	MBI-EE	4.73	0.68	4.77	0.67	3.95	1.03	4.64	0.80	3.70	1.01	4.54	0.95	3.67	0.95
	UWES^c^	3.18	1.26	3.31	1.15	3.38	1.29	3.10	1.13	3.46	1.17	3.16	1.14	3.46	1.13
	REQ-PD^c^	2.11	0.81	2.16	0.85	2.75	0.91	2.26	0.92	3.06	0.95	2.38	0.87	2.97	0.88
	Absenteeism^d^	4.93	8.70	4.40	9.62	N/A	N/A	N/A	N/A	3.64	6.70	5.23	12.10	5.88	10.57
	Presenteeism^d^	15.98	14.27	17.29	16.51	N/A	N/A	N/A	N/A	11.32	12.88	11.47	11.93	8.22	9.59
**Skills/Competencies**															
	ERSQ-C^c^	2.48	0.88	2.47	0.86	3.01	0.70	2.57	0.90	3.15	0.64	2.78	0.80	3.16	0.70
	ERSQ-A^c^	1.95	0.93	1.98	0.79	2.64	0.76	2.16	0.85	2.84	0.78	2.31	0.88	2.82	0.78
	ERSQ-SS^c^	2.02	0.91	2.15	0.82	2.70	0.73	2.32	0.88	2.79	0.82	2.38	0.86	2.73	0.86
	ERSQ-ES-GD^c^	1.79	0.54	1.81	0.52	2.43	0.56	2.04	0.59	2.53	0.58	2.04	0.55	2.58	0.52

^a^Missing data imputed by multiple imputation.

^b^Extended follow-up for intervention group only.

^c^Higher scores indicate better outcomes.

^d^In relation to the previous 3 months.

### Dropout Attrition and Handling of Missing Data

Overall, 8.0% (21/264) of participants at T2, 10.6% (28/264) of participants at T3, and 30.3% (40/132; iSMI only) of participants at T4 did not provide follow-up data for the primary outcome. A somewhat higher dropout rate was observed for the iSMI group (T2: 16/132, T3: 17/132) compared with the WLC (T2: 5/132, T3: 11/132). Thereby, groups significantly differed at T2 (χ^*2*^_1_=6.26; *P<*.05), but not at T3. Participants who did not provide follow-up data did not differ in a meaningful way from those who provided data, neither on baseline stress scores or any other baseline outcomes, with the exception of worrying (*P<*.05). Little’s overall test of randomness indicated that data were missing completely at random. Thus, multiple imputations to estimate missing values could be performed [56].

### Non-Usage Attrition

#### Intervention

Of the 132 individuals participating in the iSMI, Session 1 was completed by 122 of the participants (92.4%), Session 2 by 117 (88.6%), Session 3 by 112 (84.8%), Session 4 by 108 (81.8%), Session 5 by 103 (78.0%), Session 6 by 97 (73.5 %), Session 7 by 93 (70.5%), and the booster session by 72 (54.5%) of the participants. Because of a lack of time and changes in personal circumstances, 10 participants (7.6%) did not start the iSMI. Nine participants (6.8%) reported reasons for discontinuing the iSMI; these included lack of time (4/9), lack of motivation (3/9), technical problems (1/9), and dissatisfaction with the intervention (1/9). On average, the participants in the iSMI group completed 5.70 (SD 2.32) of the 7 sessions (81.4% of the intervention) and used the iSMI for 8.27 weeks (SD 8.54, range 0-56).

#### Text Message Support

Among the iSMI group, three-quarters (101/132, 76.5%) requested text message support via mobile phone. Of those, 43.6% (44/101) preferred light coaching and 56.4% (57/101) preferred intensive coaching at the start of the intervention.

### Other Treatment During the Trial

In the WLC condition, 37 participants (28.0%) indicated at T2 that they had received other help within the previous 7 weeks (eg, psychotherapy, health training other than the iSMI) as opposed to 24 participants (18.2%) in the iSMI condition. No significant differences were found in stress levels between those participants who had received help and those who had not.

### Primary Outcome Analyses

#### Intervention Effect

**Figure 2:**
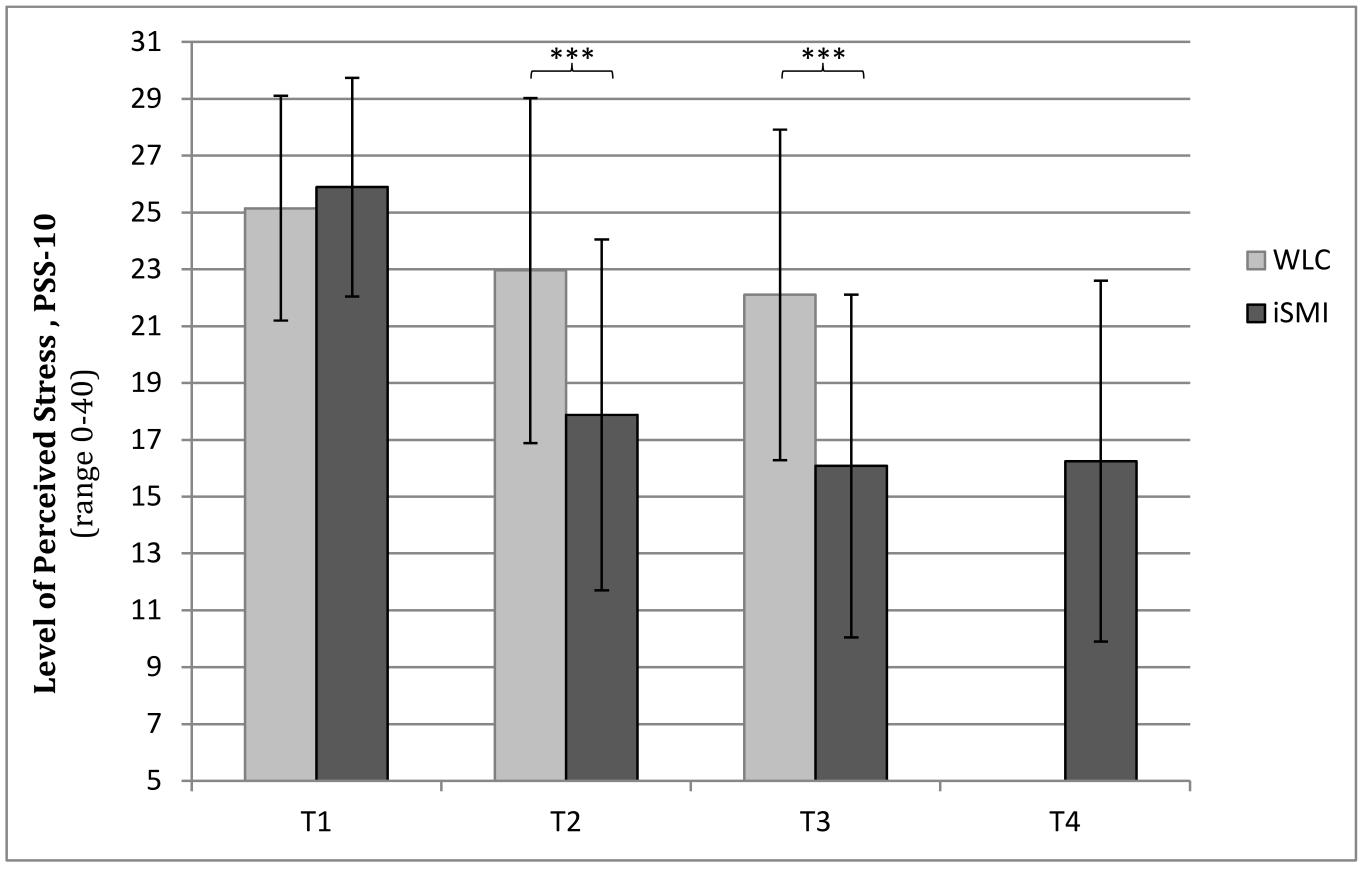
Levels of perceived stress (means and SDs) according to the PSS-10 for the iSMI and WLC groups at all assessment points for the ITT sample at pre-test (T1), post-test (T2), 6 months (T3), and 12 months (T4, iSMI only) (asterisks indicate *P*<.001).

As shown in Table 3, a significant group effect in the ANCOVA indicated that lower scores on the PSS-10 (relative to the WLC) were present for the iSMI group at T2 (*F*_1,261_=58.08, *P<*.001) and T3 (*F*_1,261_=80.17, *P<*.001). Large between-group effect sizes were observed at T2 (Cohen’s *d*=0.83; 95% CI 0.58-1.08) and T3 (*d*=1.02; 95% CI 0.76-1.27). In the intervention group, the within-group effect sizes were *d*=1.54 (95% CI 1.22-1.86) from pretest to post-test, *d*=1.92 (95% CI 1.55-2.29) from pre-test to 6-month follow-up, and *d*=1.83 (95% CI 1.45-2.21) from pre-test to 12-month follow-up. In the control group, within-group effect sizes of *d*=0.41 (95% CI 0.23-0.60) were observed from pre-test to post-test and *d*=0.60 (95% CI 0.39-0.81) from pre-test to 6-month follow-up. Figure 2 shows the PSS-10 scores for both groups at all assessment points.

#### Reliable Change

At T2, more participants in the iSMI group (81/132, 61.4%) showed reliable improvement on the PSS-10 compared with the WLC (33/132, 25.0%). A reliable deterioration was present in 1.5% (2/132) of the iSMI and 8.3% (11/132) of the WLC, whereas 37.1% (49/132; iSMI) and 66.7% (88/132; WLC) were reliably unchanged. At T3, those showing reliable improvement numbered over three-quarters (102/132, 77.3%) in the iSMI and nearly half (44/132, 33.3%) in the WLC group. Those showing reliable deterioration numbered 0.8% (1/132) in the iSMI and 6.1% (8/132) in the WLC group. No reliable change was present in 22.0% (29/132; iSMI) and 60.6% (80/132; WLC). The NNTs for reliable improvement were 2.75 (95% CI 2.11-3.96) at T2 and 2.28 (95% CI 1.83-3.01) at T3. The groups significantly differed from T1-T2 (*χ* ^*2*^_1_=37.54, *P<*.001) and from T1-T3 (Fisher’s exact=53.53, *P<*.001).

**Table 3:** Results of the ANCOVAs and Cohen’s *d* for the primary and secondary outcome measures (ITT sample) at posttest (T2) and at 6-month follow-up (T3).

Outcome		T2^a^ Between-groups effect			T3^a^ Between-groups effect		
		*d* (95% CI)	ANCOVA^b^		*d* (95% CI)	ANCOVA^b^	
			*F*_1,261_	*P*		*F*_1,261_	*P*
**Primary outcome**							
	PSS-10	0.83 (0.58-1.08)	58.08	<.001	1.02 (0.76-1.27)	80.17	<.001
**Mental health**							
	CES-D	0.64 (0.39-0.89)	34.92	<.001	0.95 (0.69-1.20)	68.29	<.001
	ISI	0.40 (0.16-0.65)	20.43	<.001	0.52 (0.27-0.76)	28.82	<.001
	HADS-A	0.68 (0.43-0.93)	49.57	<.001	0.84 (0.59-1.09)	78.94	<.001
	PSWQ-PW	0.51 (0.26-0.75)	19.74	<.001	0.63 (0.38-0.88)	31.00	<.001
	SF-12 MH	N/A	N/A		0.68 (0.43-0.93)	34.28	<.001
	SF-12 PH	N/A	N/A		0.20 (-0.05 to 0.44)	4.27	.04
**Work-related health**							
	MBI-EE	0.75 (0.50-1.00)	48.55	<.001	0.86 (0.60-1.11)	63.84	<.001
	UWES	0.23 (-0.01 to 0.47)	13.28	<.001	0.26 (0.02-0.50)	10.09	.002
	REQ-PD	0.54 (0.29-0.78)	27.45	<.001	0.75 (0.50-1.00)	52.82	<.001
	Absenteeism	N/A	N/A		0.16 (-0.08 to 0.40)	2.94	
	Presenteeism	N/A	N/A		0.01 (-0.23 to 0.25)	0.02	
**Skills/Competencies**							
	ERSQ-27-C	0.55 (0.30-0.79)	31.56	<.001	0.51 (0.27-0.76)	22.03	<.001
	ERSQ-27-A	0.60 (0.35-0.84)	30.94	<.001	0.64 (0.39-0.88)	34.96	<.001
	ERSQ-27-SS	0.47 (0.23-0.71)	29.32	<.001	0.49 (0.24-0.73)	24.56	<.001
	ERSQ-ES-GD	0.68 (0.43-0.93)	36.39	<.001	0.87 (0.61-1.12)	54.71	<.001

^a^Missing data imputed by multiple imputation.

^b^Controlling for pre-treatment scores (T1).

#### Symptom-Free Status

In this study, the cut-off score was 17.70 and below indicating a value of 2 SDs below the mean of the stressed population at T1 (mean 25.52, SD 3.91). More participants in the iSMI group met the criterion for full remission of stress symptoms compared with the WLC group at T2 (iSMI: 68/132, 51.5%; WLC: 26/132, 19.7%; χ^*2*^_1_=29.14, *P<*.001; NNT=3.14, 95% CI 2.34-4.78) and T3 (iSMI: 79/132, 59.8%; WLC: 31/132, 23.5%; χ^*2*^_1_=35.91, *P<*.001; NNT=2.75, 95% CI 2.11-3.95).

#### Completers-Only Analysis

Completers-only analyses on participants who completed all questionnaires revealed similar large effect sizes for the primary outcome at T2 (243/264, 92.0%, *d*=0.85; CI 0.59-1.11) and T3 (236/264, 89.4%, *d*=1.01; 95% CI 0.74-1.28).

### Secondary Outcome Analyses

Table 3 also shows the results of the ITT analyses for secondary outcomes for mental health, work-related health, and skills/competencies. The ANCOVAs showed highly significant between-group effects for almost all outcomes at both assessment points; all significance levels were *P<*.001 apart from work engagement at T3 (*P=*.002) and the physical health component of quality of life at T3 (*P=*.04). Between-group effects were not significant for absenteeism and presenteeism.

At T2, the majority of effect sizes were in the range of moderate (eg, *d*=0.40 for insomnia) to large (eg, *d*=0.75 for emotional exhaustion) apart from work engagement for which a small effect size was obtained (*d*=0.23). At T3, almost all effect sizes became more pronounced apart from the comprehension subscale of the ERSQ-27, which only slightly decreased (from *d*=0.55 at T2 to *d*=0.51 at T3). Thereby, large effect sizes were found at T3 for depression (*d*=0.95), anxiety (*d*=0.84), emotional exhaustion (*d*=0.86), and emotion regulation skills regarding general distress (*d*=0.87). The additional measurements taken at T3 yielded effect sizes of *d*=0.68 for the mental health component and *d*=0.20 for the physical health component of quality of life, as well as *d*=0.16 for absenteeism and *d*=0.01 for presenteeism.

#### Extended Follow-Up at 12 Months

The within-group effect size (from T1-T4) for the primary outcome PSS-10 was *d*=1.83 (95% CI 1.45-2.21). At 12-month-follow-up, improvements in all other outcome measures were also maintained at the 6-month level in the iSMI group apart from absenteeism (*P<*.05). On a descriptive level, the gains in mean days for absenteeism in the iSMI group from T1 (mean 4.93, SD 8.70) to T3 (mean 3.64, SD 6.70) could not be maintained at T4 (mean 5.88, SD 10.57). In contrast, the mean days for presenteeism in the iSMI group were almost reduced by half from T1 (mean 15.98, SD 14.27) to T4 (mean 8.22, SD 9.59).

#### Client Satisfaction

Client satisfaction with the training was high, with 92.2% (107/116) being “satisfied in an overall, general sense” (“very satisfied” or “mostly satisfied”). The majority of the participants indicated that they have received the kind of training they wanted (92.2%, 107/116; “yes, definitely” or “yes, generally”), that the training met their needs (88.8%, 103/116; “almost all […]” or “most […]”), that they are satisfied with the amount of training they received (87.9%, 102/116; “very satisfied” or “mostly satisfied”), that the training has helped them to deal more effectively with their problems (92.2%, 107/116; “yes it helped a great deal” or “yes, it helped”), and that they would use the training again if they needed to (92.2%, 107/116; “yes, definitely” or “yes, I think so”). Moreover, 90.5% (105/116) stated that they would recommend the iSMI to a friend (“yes, definitely” or “yes, I think so”).

### Explorative Analyses

#### Intervention Completion

A separate per-protocol analysis was conducted for participants who completed the intervention (≥6 sessions), which was defined as working through all of the theoretical intervention content presented up to Session 6. The ANCOVA showed significant differences between the subsample of intervention completers (97/132) and the WLC (132) with regard to perceived stress in favor of the experimental condition at T2 (*F*_1,226_=66.85, *P<*.001) and T3 (*F*_1,226_=74.70, *P<*.001) with slightly higher effect sizes at T2 (*d*=0.95; 95% CI 0.69-1.20) and T3 (*d*=1.05; 95% CI 0.79-1.31) as compared to the total iSMI sample. Within the iSMI group, we further compared intervention completers to non-completers. The ANCOVA showed a significant difference for reduction of perceived stress at T2 (*F*_1,129_=7.76, *P=*.006), but not at T3 or T4.

#### Text Message Support

There were no significant differences in the primary outcome between participants who received text messages and those who did not, nor was there any significant difference depending on the level of intensity of the individually chosen text message support.

## Discussion

### Principal Findings

The primary aim of this study was to evaluate the efficacy of a guided iSMI for employees. For this purpose, a two-arm, waitlist-controlled randomized trial was conducted. The results indicate that the training is highly effective in reducing employee stress levels in the short term (*d*=0.83) and long term (*d*=1.02) compared with the levels observed in a waitlist control group. Reduced stress levels in the iSMI group could be maintained up to 12 months. Significant medium to large between-group effects were also found for relevant secondary outcomes concerning mental health (eg, depression), work-related health (eg, emotional exhaustion), and stress-related skills (eg, emotion regulation competencies). High levels of client satisfaction and adherence were observed, and the study dropout rate was low.

The posttest effect size in stress reduction found in this study is larger than what has been found in other iSMI trials. Available RCTs on iSMI with employees show mixed between-group effects for stress at posttest, ranging from non-significant [22] to moderate effect sizes (eg, *d*=0.74; [18]). Several reasons could explain the large effect sizes found in this study. First, the intervention used a guided format. Guided iSMIs [16-18] appear to be more effective than unguided interventions [21,22,57,58], a result that is known from Web-based interventions for other mental health problems [59]. Second, the theoretical basis of the intervention was confined to two evidence-based components. Research on face-to-face interventions suggests that interventions with fewer treatment components are superior to those using more components [12]. Problem-solving training that has already been successfully introduced in other Web-based interventions to manage depressive symptoms [30] was combined here with evidence-based emotion regulation techniques including the acceptance of emotions and compassionate self-support based on the Affect Regulation Training (ART) [32]. Third, the use of mobile components to flexibly introduce training components into daily life in real time may have reinforced a regular application of the intervention exercises and therefore the efficacy of the training. The text messages were particularly popular among the participants, and given the choice of receiving the text messages, the vast majority requested this mobile component. However, we did not assess the actual engagement and future studies should compare the efficacy of the intervention with and without the mobile phone component. Fourth, the level of intervention adherence, which is regarded as leading to better treatment outcomes [60], was relatively high. Unfortunately, comparisons with adherence levels of other iSMIs are difficult as few studies have reported this information so far. Compared with the available intervention completion rates (eg, 38.5%, [14]; 44.0%, [61]; 88.2% [19]), the percentage of participants completing the intervention in this study was higher range (70.5%). Considerable efforts were undertaken to increase adherence through methods that are generally considered to be effective, including human support [62], interactive exercises [63], tailoring of the intervention [64], and reminders [65] via mobile phone. Finally, the effects may have further stabilized at T3 through the booster session, as booster sessions can be successful in maintaining treatment outcomes [66].

With regard to long-term follow-up, this work is the first study of an intervention combining Web-based and mobile components that focuses on stress reduction in employees to assess the effects compared to a control group over a longer time period (ie, 6 months). The results show that this type of intervention can have large long-term effects. Moreover, the results of this study also compare favorably to the limited existing evidence on the long-term effectiveness of iSMIs in populations other than employees, including studies finding non-significant [23] and small to moderate effect sizes (*d*=0.37 [14]) at the 6-month follow-up point. In addition, the results found for stress show similar effect sizes to those found for face-to-face interventions (*d*=0.73 [12]). Thus, iSMIs may be a useful alternative to traditional interventions.

With regard to the relationship of treatment intensity and outcome, no clear conclusions can be drawn from this study. Although intervention completers showed significantly lower stress levels at posttest, this effect was not maintained at the later follow-up points. Participants receiving text message support were also not doing significantly better as compared to those who did not receive any messages. Future research may benefit from further information on the amount of time spent on the intervention exercises in between sessions and the actual engagement with the text messages.

### Limitations

The following limitations of this trial must be acknowledged. First, for feasibility reasons, only self-report measures were assessed. Although the replacement of self-report measures with physiological measures is not recommended in occupational stress research [67], a combination of both could produce further valuable insights. Second, because this study was in the setting of indicated prevention, these results only account for participants showing relatively high baseline scores. The current sample was severely distressed and showed high baseline scores on all measures. Thus, no conclusions can be drawn regarding participants with lower stress levels (eg, in a universal prevention setting). Third, with regard to the generalizability of results, the fact that participants self-selected into the trial, the majority were female, and individuals working in the social sector were slightly overrepresented needs to be taken into consideration. Fourth, to determine the added value of the mobile component providing real-time support and encouragement, direct comparison studies would be needed comparing the intervention with and without mobile support. Fifth, the fact that the effects on physical health and work engagement were smaller than the effects on the other outcome measures demands an explanation. It is possible that Web-based interventions do not produce meaningful differences on these outcomes. Alternative explanations for the small effect sizes include that, for physical health, a rather global measure (SF-12) was applied and it may be more promising to use more specific, stress-related health measures. For work engagement, the original response categories were not adapted to the study period and the outcome measure may therefore not have been as sensitive to change. Finally, it is important to acknowledge that some improvements were also observed in the WLC group over time; in fact, this pattern has previously been found in other trials [17,31].

### Future Directions

Future research should replicate the results of this trial and investigate the moderators of outcome and adherence. It is also of interest whether the coaching time spent on each individual (up to 4 hours) could be reduced without losses in treatment effects, thereby resulting in more economical versions of iSMI. Moreover, future research should test iSMIs against the gold standard in the field (ie, face-to-face interventions) and assess which training format works best for which type of participant and under what circumstances. Although both formats may be equally effective, they may work differently on participants with varying personal characteristics and Web-based interventions may be more advantageous in terms of efficiency and costs.

### Conclusion

This trial contributes to the limited evidence base on the feasibility and efficacy of Web-based and mobile-supported stress management interventions and is among the first studies to include a longer follow-up period. The iSMI presented herein proved feasible and highly effective in improving perceived stress and other mental and work-related health indices in employees in the long term. These results indicate that this iSMI could be a valuable alternative to face-to-face trainings. Web-based interventions for coping with stress should be further evaluated as such interventions have the potential to improve the mental health of individuals on a large scale.

## Appendix

Multimedia Appendix 1 Screenshots of the intervention.

Multimedia Appendix 2 CONSORT-EHEALTH checklist V1.6.2 [67].

## Abbreviations

ANCOVA: analysis of covariance
CES-D: Center for Epidemiological Studies’ Depression Scale
CONSORT: Consolidated Standards of Reporting Trials
CSQ-8: Client Satisfaction Questionnaire
ERSQ-27-A: Emotion Regulation Skills Questionnaire – subscale acceptance
ERSQ-27-C: Emotion Regulation Skills Questionnaire – subscale comprehension,
ERSQ-ES-GD: Emotion Regulation Skills Questionnaire – Emotion specific version – subscale general distress
ERSQ-27-SS: Emotion Regulation Skills Questionnaire – subscale self-support
HADS-A: Hospital Anxiety and Depression Scales – subscale anxiety
ISI: Insomnia Severity Index
iSMI: Internet-based stress management intervention
ITT: intention-to-treat
MBI-EE: Maslach Burnout Inventory–subscale Emotional Exhaustion
NNT: number needed to treat
PSS-10: Perceived Stress Scale – 10 item version
PSWQ-PW: Penn State Worry Questionnaire-Past Week
RCT: randomized controlled trial
REQ-PD: Recovery Experience Questionnaire – subscale psychological detachment
SD: standard deviation
SF-12 MH: Short Form 12–Quality of life (mental health component)
SF-12 PH: Short Form 12–Quality of life (physical health component)
TIC-P-G: Trimbos and Institute of Medical Technology Assessment Cost Questionnaire for Psychiatry
UWES: Utrecht Work Engagement Scale
WLC: waitlist control
