# Supplementary material for: Web-Based and Mobile Stress Management Intervention for Employees: A Randomized Controlled Trial
Source: J Med Internet Res. 2016 Jan 27;18(1):e21. doi: 10.2196/jmir.5112 (PMC4749847; doi:10.2196/jmir.5112)

Supplemental material

Screenshots of the intervention

Picture 1: Starting page of the training

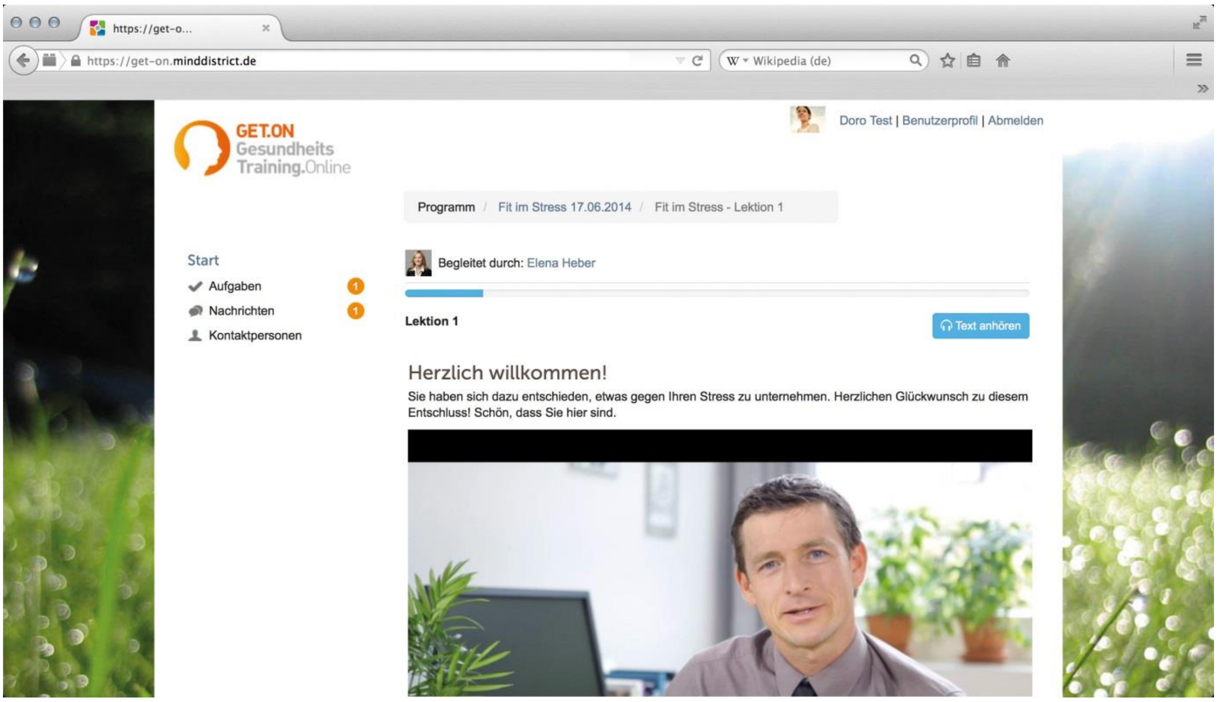

Picture 2: Six-step procedure within the problem-solving module

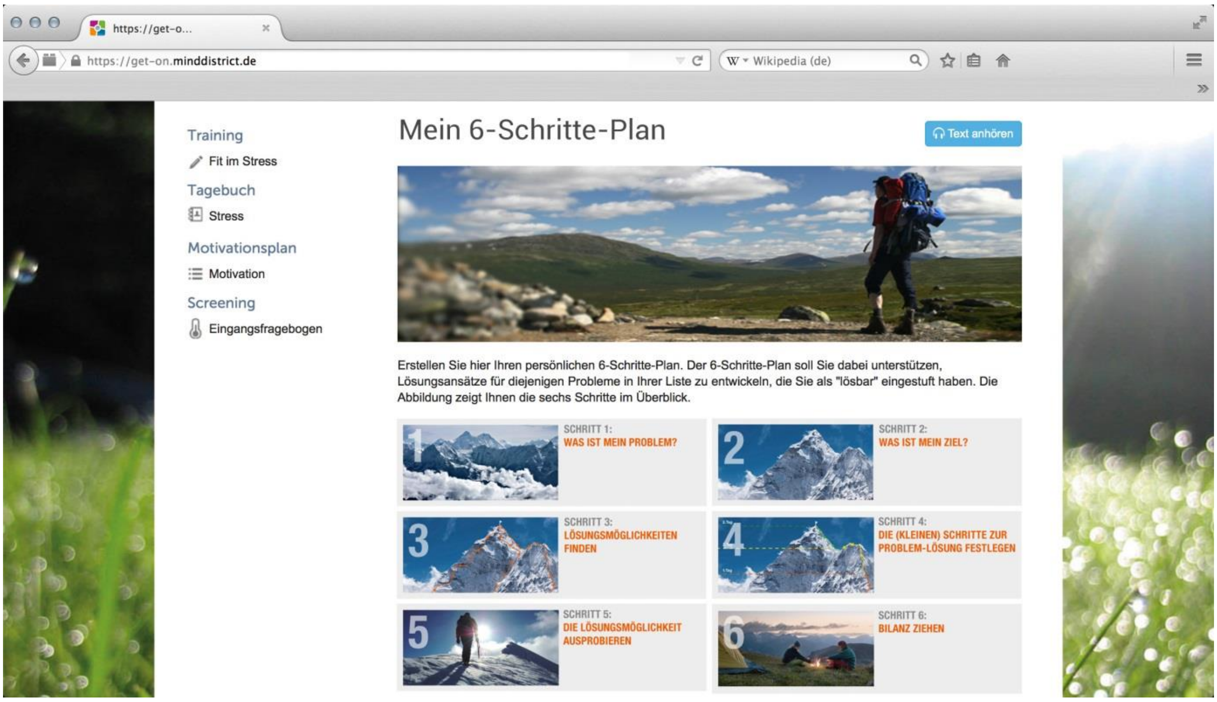

Picture 3: Exercise within the emotion regulation module

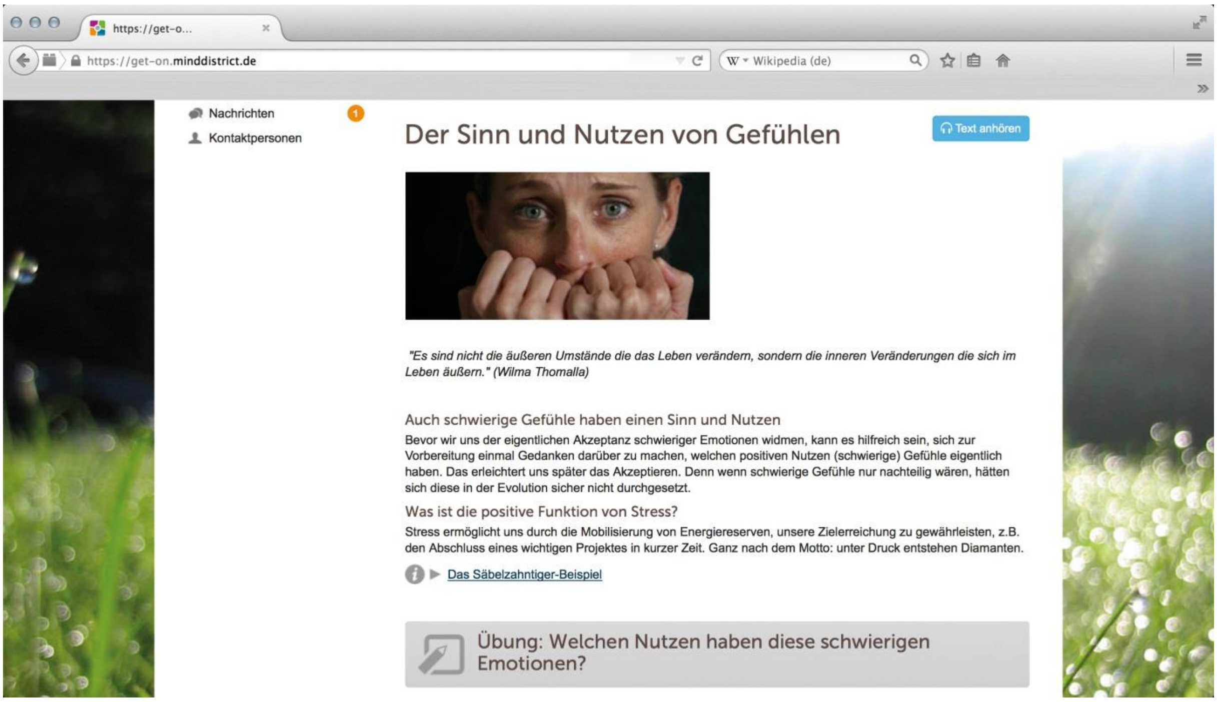

Picture 4: Planning of positive activities

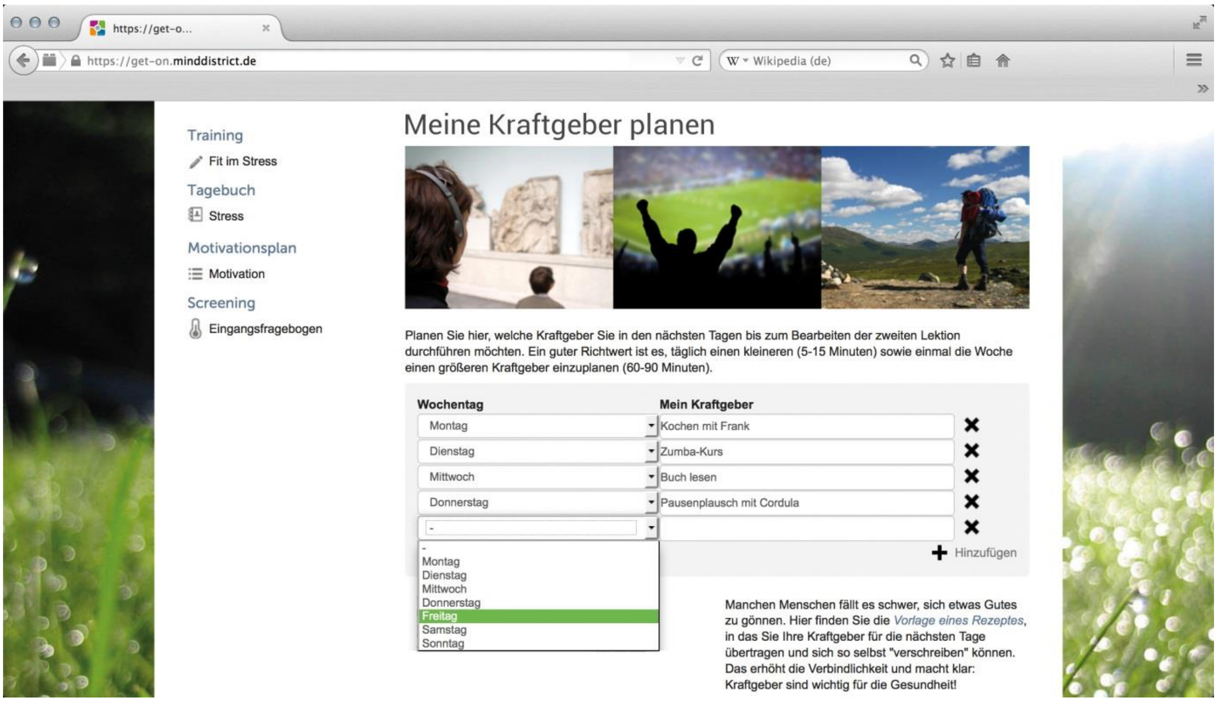

Supplement: Multimedia Appendix 1 [file jmir_v18i1e21_app1.pdf]
