# Supplementary material for: Web-Based and Mobile Stress Management Intervention for Employees: A Randomized Controlled Trial
Source: J Med Internet Res. 2016 Jan 27;18(1):e21. doi: 10.2196/jmir.5112 (PMC4749847; doi:10.2196/jmir.5112)
Supplement: Multimedia Appendix 2 [file jmir_v18i1e21_app2.pdf]

CONSORT-EHEALTH (V 1.6.1) - Submission/Publication Form

The CONSORT-EHEALTH checklist is intended for authors of randomized trials evaluating web-based and Internet-based applications/interventions, including mobile interventions, electronic games (incl multiplayer games), social media, certain telehealth applications, and other interactive and/or networked electronic applications. Some of the items (e.g. all subitems under item 5 - description of the intervention) may also be applicable for other study designs.

The goal of the CONSORT EHEALTH checklist and guideline is to be
a) a guide for reporting for authors of RCTs,
b) to form a basis for appraisal of an ehealth trial (in terms of validity)

CONSORT-EHEALTH items/subitems are MANDATORY reporting items for studies published in the Journal of Medical Internet Research and other journals / scientific societies endorsing the checklist.

Items numbered 1., 2., 3., 4a., 4b etc are original CONSORT or CONSORT-NPT (non-pharmacologic treatment) items.
Items with Roman numerals (i., ii, iii, iv etc.) are CONSORT-EHEALTH extensions/clarifications.

As the CONSORT-EHEALTH checklist is still considered in a formative stage, we would ask that you also RATE ON A SCALE OF 1-5 how important/useful you feel each item is FOR THE PURPOSE OF THE CHECKLIST and reporting guideline (optional).

Mandatory reporting items are marked with a red *.
In the textboxes, either copy & paste the relevant sections from your manuscript into this form - please include any quotes from your manuscript in QUOTATION MARKS,
or answer directly by providing additional information not in the manuscript, or elaborating on why the item was not relevant for this study.

YOUR ANSWERS WILL BE PUBLISHED AS A SUPPLEMENTARY FILE TO YOUR PUBLICATION IN JMIR AND ARE CONSIDERED PART OF YOUR PUBLICATION (IF ACCEPTED).
Please fill in these questions diligently. Information will not be copyedited, so please use proper spelling and grammar, use correct capitalization, and avoid abbreviations.

DO NOT FORGET TO SAVE AS PDF _AND_ CLICK THE SUBMIT BUTTON SO YOUR ANSWERS ARE IN OUR DATABASE !!!

Citation Suggestion (if you append the pdf as Appendix we suggest to cite this paper in the caption):
Eysenbach G, CONSORT-EHEALTH Group
CONSORT-EHEALTH: Improving and Standardizing Evaluation Reports of Web-based and Mobile Health Interventions
J Med Internet Res 2011;13(4):e126
URL: http://www.jmir.org/2011/4/e126/
doi: 10.2196/jmir.1923
PMID: 22209829

* Erforderlich

Your name *
First Last

Elena Heber

Primary Affiliation (short), City, Country *

University of Toronto, Toronto, Canada

Leuphana University Luene

Your e-mail address *

abc@gmail.com

ber@inkubator.leuphana.d

Title of your manuscript *

Provide the (draft) title of your manuscript.

Web-based and mobile stress management intervention for employees: results of a randomised controlled trial

Article Preparation Status/Stage *

At which stage in your article preparation are you currently (at the time you fill in this form)

- ○ not submitted yet - in early draft status
- ◉ not submitted yet - in late draft status, just before submission
- ○ submitted to a journal but not reviewed yet
- ○ submitted to a journal and after receiving initial reviewer comments
- ○ submitted to a journal and accepted, but not published yet
- ○ published
- ○ Sonstiges:

Journal *

If you already know where you will submit this paper (or if it is already submitted), please provide the journal name (if it is not JMIR, provide the journal name under "other")

- ○ not submitted yet / unclear where I will submit this
- ◉ Journal of Medical Internet Research (JMIR)
- ○ Sonstiges:

Manuscript tracking number *

If this is a JMIR submission, please provide the manuscript tracking number under "other" (The ms tracking number can be found in the submission acknowledgement email, or when you login as author in JMIR. If the paper is already published in JMIR, then the ms tracking number is the four-digit number at the end of the DOI, to be found at the bottom of each published article in JMIR)

- ◉ no ms number (yet) / not (yet) submitted to / published in JMIR
- ○ Sonstiges:

TITLE AND ABSTRACT

1a) TITLE: Identification as a randomized trial in the title

1a) Does your paper address CONSORT item 1a? *

I.e does the title contain the phrase "Randomized Controlled Trial"? (if not, explain the reason under "other")

- (x) yes
- ( ) Sonstiges:

1a-i) Identify the mode of delivery in the title

Identify the mode of delivery. Preferably use "web-based" and/or "mobile" and/or "electronic game" in the title. Avoid ambiguous terms like "online", "virtual", "interactive". Use "Internet-based" only if Intervention includes non-web-based Internet components (e.g. email), use "computer-based" or "electronic" only if offline products are used. Use "virtual" only in the context of "virtual reality" (3-D worlds). Use "online" only in the context of "online support groups". Complement or substitute product names with broader terms for the class of products (such as "mobile" or "smart phone" instead of "iphone"), especially if the application runs on different platforms.

	1	2	3	4	5	
subitem not at all important	( )	( )	( )	( )	( )	essential

Does your paper address subitem 1a-i? *

Copy and paste relevant sections from manuscript title (include quotes in quotation marks "like this" to indicate direct quotes from your manuscript), or elaborate on this item by providing additional information not in the ms, or briefly explain why the item is not applicable/relevant for your study

"Web-based and mobile stress management intervention for employees: results of a randomised controlled trial"

1a-ii) Non-web-based components or important co-interventions in title

Mention non-web-based components or important co-interventions in title, if any (e.g., "with telephone support").

	1	2	3	4	5	
subitem not at all important	( )	( )	( )	( )	( )	essential

Does your paper address subitem 1a-ii?

Copy and paste relevant sections from manuscript title (include quotes in quotation marks "like this" to indicate direct quotes from your manuscript), or elaborate on this item by providing additional information not in the ms, or briefly explain why the item is not applicable/relevant for your study

There are no non-web-based components.

1a-iii) Primary condition or target group in the title

Mention primary condition or target group in the title, if any (e.g., "for children with Type I Diabetes") Example: A Web-based and Mobile Intervention with Telephone Support for Children with Type I Diabetes: Randomized Controlled Trial

	1	2	3	4	5	
subitem not at all important	○	○	○	○	○	essential

Does your paper address subitem 1a-iii? *

Copy and paste relevant sections from manuscript title (include quotes in quotation marks "like this" to indicate direct quotes from your manuscript), or elaborate on this item by providing additional information not in the ms, or briefly explain why the item is not applicable/relevant for your study

"for employees"

1b) ABSTRACT: Structured summary of trial design, methods, results, and conclusions

NPT extension: Description of experimental treatment, comparator, care providers, centers, and blinding status.

1b-i) Key features/functionalities/components of the intervention and comparator in the METHODS section of the ABSTRACT

Mention key features/functionalities/components of the intervention and comparator in the abstract. If possible, also mention theories and principles used for designing the site. Keep in mind the needs of systematic reviewers and indexers by including important synonyms. (Note: Only report in the abstract what the main paper is reporting. If this information is missing from the main body of text, consider adding it)

	1	2	3	4	5	
subitem not at all important	○	○	○	○	○	essential

Does your paper address subitem 1b-i? *

Copy and paste relevant sections from the manuscript abstract (include quotes in quotation marks "like this" to indicate direct quotes from your manuscript), or elaborate on this item by providing additional information not in the ms, or briefly explain why the item is not applicable/relevant for your study

"The intervention (GET.ON Stress) was based on Lazarus' transactional model of stress, consisted of seven sessions and applied both well-established problem solving and more recently developed emotion regulation strategies. Participants also had the opportunity to request automatic text messages on their mobile phone along with the iSMI."

"...randomly assigned to an intervention (iSMI) or waitlist control group (WLC)."

1b-ii) Level of human involvement in the METHODS section of the ABSTRACT

Clarify the level of human involvement in the abstract, e.g., use phrases like "fully automated" vs. "therapist/nurse/care provider/physician-assisted" (mention number and expertise of providers involved, if any). (Note: Only report in the abstract what the main paper is reporting. If this information is missing from the main body of text, consider adding it)

	1	2	3	4	5	
subitem not at all important	○	○	○	○	○	essential

Does your paper address subitem 1b-ii?

Copy and paste relevant sections from the manuscript abstract (include quotes in quotation marks "like this" to indicate direct quotes from your manuscript), or elaborate on this item by providing additional information not in the ms, or briefly explain why the item is not applicable/relevant for your study

"Participants received written feedback on every completed session from an e-coach."

1b-iii) Open vs. closed, web-based (self-assessment) vs. face-to-face assessments in the METHODS section of the ABSTRACT

Mention how participants were recruited (online vs. offline), e.g., from an open access website or from a clinic or a closed online user group (closed usergroup trial), and clarify if this was a purely web-based trial, or there were face-to-face components (as part of the intervention or for assessment). Clearly say if outcomes were self-assessed through questionnaires (as common in web-based trials). Note: In traditional offline trials, an open trial (open-label trial) is a type of clinical trial in which both the researchers and participants know which treatment is being administered. To avoid confusion, use "blinded" or "unblinded" to indicated the level of blinding instead of "open", as "open" in web-based trials usually refers to "open access" (i.e. participants can self-enrol). (Note: Only report in the abstract what the main paper is reporting. If this information is missing from the main body of text, consider adding it)

	1	2	3	4	5	
subitem not at all important	○	○	○	○	○	essential

Does your paper address subitem 1b-iii?

Copy and paste relevant sections from the manuscript abstract (include quotes in quotation marks "like this" to indicate direct quotes from your manuscript), or elaborate on this item by providing additional information not in the ms, or briefly explain why the item is not applicable/relevant for your study

"Web-based self-report assessments for both groups were scheduled at baseline, at seven weeks, and at six months."

1b-iv) RESULTS section in abstract must contain use data

Report number of participants enrolled/assessed in each group, the use/uptake of the intervention (e.g., attrition/adherence metrics, use over time, number of logins etc.), in addition to primary/secondary outcomes. (Note: Only report in the abstract what the main paper is reporting. If this information is missing from the main body of text, consider adding it)

	1	2	3	4	5	
subitem not at all important	◯	◯	◯	◯	◯	essential

Does your paper address subitem 1b-iv?

Copy and paste relevant sections from the manuscript abstract (include quotes in quotation marks "like this" to indicate direct quotes from your manuscript), or elaborate on this item by providing additional information not in the ms, or briefly explain why the item is not applicable/relevant for your study

"An intention-to-treat analysis of covariance revealed significantly large effect differences between iSMI and WLC groups for perceived stress at post-test ($F(1,261)=58.08$, $P<.001$; Cohen's $d=0.83$) and at the 6-month follow-up ($F(1,261)=80.17$, $P<.001$; Cohen's $d=1.02$). The effects in the iSMI group were maintained at the 12-month follow-up."

1b-v) CONCLUSIONS/DISCUSSION in abstract for negative trials

Conclusions/Discussions in abstract for negative trials: Discuss the primary outcome - if the trial is negative (primary outcome not changed), and the intervention was not used, discuss whether negative results are attributable to lack of uptake and discuss reasons. (Note: Only report in the abstract what the main paper is reporting. If this information is missing from the main body of text, consider adding it)

	1	2	3	4	5	
subitem not at all important	◯	◯	◯	◯	◯	essential

Does your paper address subitem 1b-v?

Copy and paste relevant sections from the manuscript abstract (include quotes in quotation marks "like this" to indicate direct quotes from your manuscript), or elaborate on this item by providing additional information not in the ms, or briefly explain why the item is not applicable/relevant for your study

Not applicable

INTRODUCTION

2a) In INTRODUCTION: Scientific background and explanation of rationale

2a-i) Problem and the type of system/solution

Describe the problem and the type of system/solution that is object of the study: intended as stand-alone intervention vs. incorporated in broader health care program? Intended for a particular patient population? Goals of the intervention, e.g., being more cost-effective to other interventions, replace or complement other solutions? (Note: Details about the intervention are provided in "Methods" under 5)

	1	2	3	4	5	
subitem not at all important	○	○	○	○	○	essential

Does your paper address subitem 2a-i? *

Copy and paste relevant sections from the manuscript (include quotes in quotation marks "like this" to indicate direct quotes from your manuscript), or elaborate on this item by providing additional information not in the ms, or briefly explain why the item is not applicable/relevant for your study

"Stress and related adverse outcomes for physical and mental health are highly prevalent and pose a major threat to public health. Individuals with high stress levels face various negative consequences of stress including sleeping problems [citation], burnout [citation], an increased risk of depression, anxiety [citation], and coronary heart disease [citation]. According to a recent survey [citation], 31% of U.S. employees feel tense or stressed on a daily basis. Meanwhile, 64 % report receiving insufficient stress management resources from their employers.

In recent years, web- and mobile-based interventions for coping with work-related stress have emerged. The advantages attributed to web-based interventions include the potential for large-scale delivery, 24/7 availability, low costs and a low access threshold [citation]. Moreover, a recent meta-analysis reveals an equivalence between face-to-face and internet-based guided cognitive behavioural therapy [citation]. For populations experiencing high levels of work-related stress, web-based interventions can be an appealing method for flexibly integrating the stress management exercises into daily life. In particular, mobile behavioural intervention technologies for mental health offer the potential to deliver training components in real time and real world [citation]. Internet-based interventions may also reach those who are unwilling to participate in traditional face-to-face interventions [citation]. "

2a-ii) Scientific background, rationale: What is known about the (type of) system

Scientific background, rationale: What is known about the (type of) system that is the object of the study (be sure to discuss the use of similar systems for other conditions/diagnoses, if appropiate), motivation for the study, i.e. what are the reasons for and what is the context for this specific study, from which stakeholder viewpoint is the study performed, potential impact of findings [2]. Briefly justify the choice of the comparator.

	1	2	3	4	5	
subitem not at all important	○	○	○	○	○	essential

Does your paper address subitem 2a-ii? *

Copy and paste relevant sections from the manuscript (include quotes in quotation marks "like this" to indicate direct quotes from your manuscript), or elaborate on this item by providing additional information not in the ms, or briefly explain why the item is not applicable/relevant for your study

"Face-to-face trainings on stress management have been proven to be effective [citation]. However, the evidence base for internet-based stress management interventions (iSMIs) remains inconclusive, as only a limited number of randomised controlled trials (RCTs) have been conducted. Some of these studies showed a significant moderate reduction of stress for web-based interventions compared with a waitlist group (e.g., [citation]), a no-treatment group (e.g., [citation]) and an attention control group (e.g., [citation]). Other studies did not find beneficial between-group effects for stress at post-test (e.g., [citation]). For instance, Wolever et al. [citation] found an effect size of d=0.74 for reduced stress for a guided mindfulness at work intervention, whereas Wiegand et al. [citation] did not find significant between-group effects for an unguided comprehensive web program that included olfactory care products for women. This lack of conclusiveness of iSMIs also applies to other mental health indicators, such as depression. The differences in effectiveness may result from variations in the type and length of interventions studied, the usage of guidance, the outcomes, the measurements, or the setting. Likewise, little is known about the long-term efficacy of iSMIs. Two RCTs have investigated the efficacy of iSMIs at the 6-month follow-up relative to a control group finding a non-significant effect for stress reduction in students [citation] and a small to moderate effect for the general population [citation]. An extended follow-up conducted by Ruwaard et al. [citation] over a three year period yielded beneficial results for reducing stress. However, no RCTs investigating an intervention combining web-based and mobile components with a focus on stress reduction have addressed employees as a target group relative to a control group in long-term follow-ups (e.g., 6 months)."

2b) In INTRODUCTION: Specific objectives or hypotheses

Does your paper address CONSORT subitem 2b? *

Copy and paste relevant sections from the manuscript (include quotes in quotation marks "like this" to indicate direct quotes from your manuscript), or elaborate on this item by providing additional information not in the ms, or briefly explain why the item is not applicable/relevant for your study

"This paper presents the results of a randomised waitlist-controlled trial to investigate the efficacy of a newly developed iSMI that includes mobile components for reducing stress in employees with elevated stress levels. We assessed whether the primary outcome perceived stress (PSS-10) showed any differences between the intervention (iSMI) group and a waitlist control (WLC) group at post-test and at 6-month follow-up. Among the secondary outcomes, selected mental- and work-related health indicators often perceived to arise due to chronic stress, such as depression, anxiety, and emotional exhaustion were also considered."

METHODS

3a) Description of trial design (such as parallel, factorial) including allocation ratio

Does your paper address CONSORT subitem 3a? *

Copy and paste relevant sections from the manuscript (include quotes in quotation marks "like this" to indicate direct quotes from your manuscript), or elaborate on this item by providing additional information not in the ms, or briefly explain why the item is not applicable/relevant for your study

"Using a 2-arm randomised controlled design, 264 participants were randomly allocated (at a ratio of 1:1 and a block size of 2) to the iSMI or to a WLC group."

3b) Important changes to methods after trial commencement (such as eligibility criteria), with reasons

Does your paper address CONSORT subitem 3b? *

Copy and paste relevant sections from the manuscript (include quotes in quotation marks "like this" to indicate direct quotes from your manuscript), or elaborate on this item by providing additional information not in the ms, or briefly explain why the item is not applicable/relevant for your study

No changes occurred.

3b-i) Bug fixes, Downtimes, Content Changes

Bug fixes, Downtimes, Content Changes: ehealth systems are often dynamic systems. A description of changes to methods therefore also includes important changes made on the intervention or comparator during the trial (e.g., major bug fixes or changes in the functionality or content) (5-iii) and other "unexpected events" that may have influenced study design such as staff changes, system failures/downtimes, etc. [2].

	1	2	3	4	5	
subitem not at all important	○	○	○	○	○	essential

Does your paper address subitem 3b-i?

Copy and paste relevant sections from the manuscript (include quotes in quotation marks "like this" to indicate direct quotes from your manuscript), or elaborate on this item by providing additional information not in the ms, or briefly explain why the item is not applicable/relevant for your study

No major bug fixes or changes in the functionality or content occurred.

4a) Eligibility criteria for participants

Does your paper address CONSORT subitem 4a? *

Copy and paste relevant sections from the manuscript (include quotes in quotation marks "like this" to indicate direct quotes from your manuscript), or elaborate on this item by providing additional information not in the ms, or briefly explain why the item is not applicable/relevant for your study

information not in the ms, or briefly explain why the item is not applicable/relevant for your study

"Participants 18 years and older were included if they were currently employed and scored 22 or above on the Perceived Stress Scale (PSS-10) indicating one standard deviation (SD) above the mean in a large working population to choose participants with an elevated level of stress [citation]. Any applicants who were at risk of suicide (Beck Suicide Item > 1; [citation]) or previously diagnosed with dissociative or psychotic symptoms were excluded."

4a-i) Computer / Internet literacy

Computer / Internet literacy is often an implicit "de facto" eligibility criterion - this should be explicitly clarified.

	1	2	3	4	5	
subitem not at all important	◯	◯	◯	◯	◯	essential

Does your paper address subitem 4a-i?

Copy and paste relevant sections from the manuscript (include quotes in quotation marks "like this" to indicate direct quotes from your manuscript), or elaborate on this item by providing additional information not in the ms, or briefly explain why the item is not applicable/relevant for your study

"Those interested in participating had to provide an e-mail address..."

4a-ii) Open vs. closed, web-based vs. face-to-face assessments:

Open vs. closed, web-based vs. face-to-face assessments: Mention how participants were recruited (online vs. offline), e.g., from an open access website or from a clinic, and clarify if this was a purely web-based trial, or there were face-to-face components (as part of the intervention or for assessment), i.e., to what degree got the study team to know the participant. In online-only trials, clarify if participants were quasi-anonymous and whether having multiple identities was possible or whether technical or logistical measures (e.g., cookies, email confirmation, phone calls) were used to detect/prevent these.

	1	2	3	4	5	
subitem not at all important	◯	◯	◯	◯	◯	essential

Does your paper address subitem 4a-ii? *

Copy and paste relevant sections from the manuscript (include quotes in quotation marks "like this" to indicate direct quotes from your manuscript), or elaborate on this item by providing additional information not in the ms, or briefly explain why the item is not applicable/relevant for your study

"Recruitment occurred from January to October 2013 in the general working population through newspaper articles, announcements by a ministry of education and advertisements in the membership magazine of a large German health insurance company. Those interested in participating had to provide an e-mail address and a first and last name which could be pseudonyms if desired. Using the e-mail addresses provided individuals received a link to the screening questionnaire. Provided that they were eligible, applicants had to submit their signed informed consent via post or scanned via e-mail and complete the baseline assessment."

"All questionnaires were self-assessed online [...]."

4a-iii) Information giving during recruitment

Information given during recruitment. Specify how participants were briefed for recruitment and in the informed consent procedures (e.g., publish the informed consent documentation as appendix, see also item X26), as this information may have an effect on user self-selection, user expectation and may also bias results.

	1	2	3	4	5	
subitem not at all important	◯	◯	◯	◯	◯	essential

Does your paper address subitem 4a-iii?

Copy and paste relevant sections from the manuscript (include quotes in quotation marks "like this" to indicate direct quotes from your manuscript), or elaborate on this item by providing additional information not in the ms, or briefly explain why the item is not applicable/relevant for your study

"Provided that they were eligible, applicants had to submit their signed informed consent via post or scanned via e-mail and complete the baseline assessment."

4b) Settings and locations where the data were collected

Does your paper address CONSORT subitem 4b? *

Copy and paste relevant sections from the manuscript (include quotes in quotation marks "like this" to indicate direct quotes from your manuscript), or elaborate on this item by providing additional information not in the ms, or briefly explain why the item is not applicable/relevant for your study

"All questionnaires were self-assessed online [...]."

4b-i) Report if outcomes were (self-)assessed through online questionnaires

Clearly report if outcomes were (self-)assessed through online questionnaires (as common in web-based trials) or otherwise.

	1	2	3	4	5	
subitem not at all important	◯	◯	◯	◯	◯	essential

Does your paper address subitem 4b-i? *

Copy and paste relevant sections from the manuscript (include quotes in quotation marks "like this" to indicate direct quotes from your manuscript), or elaborate on this item by providing additional information not in the ms, or briefly explain why the item is not applicable/relevant for your study

"All questionnaires were self-assessed online at baseline (T1), seven weeks (T2, post-treatment), 6 months (T3) and 12 months (T4, iSMI group only) after randomisation."

4b-ii) Report how institutional affiliations are displayed

Report how institutional affiliations are displayed to potential participants [on ehealth media], as affiliations with prestigious hospitals or universities may affect volunteer rates, use, and reactions with regards to an intervention.(Not a required item – describe only if this may bias results)

	1	2	3	4	5	
subitem not at all important	◯	◯	◯	◯	◯	essential

Does your paper address subitem 4b-ii?

Copy and paste relevant sections from the manuscript (include quotes in quotation marks "like this" to indicate direct quotes from your manuscript), or elaborate on this item by providing additional information not in the ms, or briefly explain why the item is not applicable/relevant for your study

"Recruitment occurred [...] through newspaper articles, announcements by a ministry of education and advertisements in the membership magazine of a large German health insurance company. "

5) The interventions for each group with sufficient details to allow replication, including how and when they were actually administered

5-i) Mention names, credential, affiliations of the developers, sponsors, and owners

Mention names, credential, affiliations of the developers, sponsors, and owners [6] (if authors/evaluators are owners or developer of the software, this needs to be declared in a "Conflict of interest" section or mentioned elsewhere in the manuscript).

	1	2	3	4	5	
subitem not at all important	○	○	○	○	○	essential

Does your paper address subitem 5-i?

Copy and paste relevant sections from the manuscript (include quotes in quotation marks "like this" to indicate direct quotes from your manuscript), or elaborate on this item by providing additional information not in the ms, or briefly explain why the item is not applicable/relevant for your study

The intervention was developed at the Leuphana University Lueneburg, Germany by the authors of this article using a web-based platform (https://get-on.minddistrict.de/).

5-ii) Describe the history/development process

Describe the history/development process of the application and previous formative evaluations (e.g., focus groups, usability testing), as these will have an impact on adoption/use rates and help with interpreting results.

	1	2	3	4	5	
subitem not at all important	○	○	○	○	○	essential

Does your paper address subitem 5-ii?

Copy and paste relevant sections from the manuscript (include quotes in quotation marks "like this" to indicate direct quotes from your manuscript), or elaborate on this item by providing additional information not in the ms, or briefly explain why the item is not applicable/relevant for your study

The intervention was piloted in a small number of employees prior to the study asking about the participant's opinion of the intervention on several aspects (e.g., structure of the program, usefulness of specific intervention components, e-coaching, time spent on the intervention). This information was used to improve the intervention prior to the start of the study.

5-iii) Revisions and updating

Revisions and updating. Clearly mention the date and/or version number of the application/intervention (and comparator, if applicable) evaluated, or describe whether the intervention underwent major changes during the evaluation process, or whether the development and/or content was "frozen" during the trial. Describe dynamic components such as news feeds or changing content which may have an impact on the replicability of the intervention (for unexpected events see item 3b).

	1	2	3	4	5	
subitem not at all important	◯	◯	◯	◯	◯	essential

Does your paper address subitem 5-iii?

Copy and paste relevant sections from the manuscript (include quotes in quotation marks "like this" to indicate direct quotes from your manuscript), or elaborate on this item by providing additional information not in the ms, or briefly explain why the item is not applicable/relevant for your study

The intervention content was "frozen" during the trial.

5-iv) Quality assurance methods

Provide information on quality assurance methods to ensure accuracy and quality of information provided [1], if applicable.

	1	2	3	4	5	
subitem not at all important	◯	◯	◯	◯	◯	essential

Does your paper address subitem 5-iv?

Copy and paste relevant sections from the manuscript (include quotes in quotation marks "like this" to indicate direct quotes from your manuscript), or elaborate on this item by providing additional information not in the ms, or briefly explain why the item is not applicable/relevant for your study

5-v) Ensure replicability by publishing the source code, and/or providing screenshots/screen-capture video, and/or providing flowcharts of the algorithms used

Ensure replicability by publishing the source code, and/or providing screenshots/screen-capture video, and/or providing flowcharts of the algorithms used. Replicability (i.e., other researchers should in principle be able to replicate the study) is a hallmark of scientific reporting.

	1	2	3	4	5	
subitem not at all important	◯	◯	◯	◯	◯	essential

Does your paper address subitem 5-v?

Copy and paste relevant sections from the manuscript (include quotes in quotation marks "like this" to indicate direct quotes from your manuscript), or elaborate on this item by providing additional information not in the ms, or briefly explain why the item is not applicable/relevant for your study

Screenshots of the intervention are included in the supplemental material.

5-vi) Digital preservation

Digital preservation: Provide the URL of the application, but as the intervention is likely to change or disappear over the course of the years; also make sure the intervention is archived (Internet Archive, webcitation.org, and/or publishing the source code or screenshots/videos alongside the article). As

pages behind login screens cannot be archived, consider creating demo pages which are accessible without login.

	1	2	3	4	5	
subitem not at all important	○	○	○	○	○	essential

Does your paper address subitem 5-vi?

Copy and paste relevant sections from the manuscript (include quotes in quotation marks "like this" to indicate direct quotes from your manuscript), or elaborate on this item by providing additional information not in the ms, or briefly explain why the item is not applicable/relevant for your study

The log-in page of the web-based intervention can be found here: http://www.webcitation.org/6bOOmekws.

Screenshots of the intervention are included in the supplemental material.

5-vii) Access

Access: Describe how participants accessed the application, in what setting/context, if they had to pay (or were paid) or not, whether they had to be a member of specific group. If known, describe how participants obtained "access to the platform and Internet" [1]. To ensure access for editors/reviewers /readers, consider to provide a "backdoor" login account or demo mode for reviewers/readers to explore the application (also important for archiving purposes, see vi).

	1	2	3	4	5	
subitem not at all important	○	○	○	○	○	essential

Does your paper address subitem 5-vii? *

Copy and paste relevant sections from the manuscript (include quotes in quotation marks "like this" to indicate direct quotes from your manuscript), or elaborate on this item by providing additional information not in the ms, or briefly explain why the item is not applicable/relevant for your study

"The program [...] was presented on a secured web-based platform. Upon activation of the account through the research team, participants used their e-mail address and a self-chosen password to log on."

5-viii) Mode of delivery, features/functionalities/components of the intervention and comparator, and the theoretical framework

Describe mode of delivery, features/functionalities/components of the intervention and comparator, and the theoretical framework [6] used to design them (instructional strategy [1], behaviour change techniques, persuasive features, etc., see e.g., [7, 8] for terminology). This includes an in-depth description of the content (including where it is coming from and who developed it) [1]," whether [and how] it is tailored to individual circumstances and allows users to track their progress and receive feedback" [6]. This also includes a description of communication delivery channels and – if computer-mediated communication is a component – whether communication was synchronous or asynchronous [6]. It also includes information on presentation strategies [1], including page design principles, average amount of text on pages, presence of hyperlinks to other resources, etc. [1].

	1	2	3	4	5	
subitem not at all important	○	○	○	○	○	essential

Does your paper address subitem 5-viii? *

Copy and paste relevant sections from the manuscript (include quotes in quotation marks "like this" to indicate direct quotes from your manuscript), or elaborate on this item by providing additional information not in the ms, or briefly explain why the item is not applicable/relevant for your study

"The iSMI GET.ON Stress is based on Lazarus' transactional model of stress [citation]. This intervention applied both well-established problem solving and more recently developed emotion regulation strategies. Important principles for health behaviour change such as goal setting, action planning and coping planning were followed. The iSMI consisted of seven sessions and a booster session provided four weeks after training completion. Following psycho-education (session 1), the participants learned a six-step-procedure to systematically solve problems (sessions 2-3). In sessions 4-6, the participants were introduced to emotion regulation techniques (muscle- and breathing relaxation, acceptance of negative emotions, and self-support in difficult situations). Session 7 included a plan for the future. The iSMI was specifically tailored to employees; this was reflected in the wording of the intervention, the example characters provided throughout the training as well as in optional short informational material related to typical stress-related topics (e.g., psychological detachment from work, time management, sleep hygiene, worrying, and organisation of breaks during work) which was provided alongside the intervention. The application of exercises was strongly recommended. The participants were advised to complete 1-2 sessions per week. The program included interactive exercises, audio/video files, and downloadable material and was presented on a secured web-based platform. [...] Within 48 hours after session completion, an e-coach provided approximately three quarters of a page of written, non-therapeutic feedback intended to increase adherence and motivation. The e-coaches reported that the average time spent per feedback was 30 minutes. In the event of non-completion of a session they also sent reminders.[...] The participants could receive automatic text messages on their mobile phone along with the iSMI (e.g., short relaxation exercises: "Relax your muscles in your hands and arms for 3 seconds now. Follow your breathing and each time you breathe out, relax a little more."), given the choice of either light (1 text message every other day) or intensive support (2-3 text messages per day). "

5-ix) Describe use parameters

Describe use parameters (e.g., intended "doses" and optimal timing for use). Clarify what instructions or recommendations were given to the user, e.g., regarding timing, frequency, heaviness of use, if any, or was the intervention used ad libitum.

	1	2	3	4	5	
subitem not at all important	○	○	○	○	○	essential

Does your paper address subitem 5-ix?

Copy and paste relevant sections from the manuscript (include quotes in quotation marks "like this" to indicate direct quotes from your manuscript), or elaborate on this item by providing additional information not in the ms, or briefly explain why the item is not applicable/relevant for your study

"The application of exercises was strongly recommended. The participants were advised to complete 1-2 sessions per week."

5-x) Clarify the level of human involvement

Clarify the level of human involvement (care providers or health professionals, also technical assistance) in the e-intervention or as co-intervention (detail number and expertise of professionals involved, if any, as well as "type of assistance offered, the timing and frequency of the support, how it is initiated, and the medium by which the assistance is delivered". It may be necessary to distinguish between the level of human involvement required for the trial, and the level of human involvement required for a routine application outside of a RCT setting (discuss under item 21 – generalizability).

	1	2	3	4	5	
subitem not at all important	○	○	○	○	○	essential

Does your paper address subitem 5-x?

Copy and paste relevant sections from the manuscript (include quotes in quotation marks "like this" to indicate direct quotes from your manuscript), or elaborate on this item by providing additional information not in the ms, or briefly explain why the item is not applicable/relevant for your study

"Within 48 hours after session completion, an e-coach provided approximately three quarters of a page of written, non-therapeutic feedback intended to increase adherence and motivation. The e-coaches reported that the average time spent per feedback was 30 minutes. In the event of non-completion of a session they also sent reminders. Each e-coach had a degree in psychology and followed a standardised manual on feedback writing."

5-xi) Report any prompts/reminders used

Report any prompts/reminders used: Clarify if there were prompts (letters, emails, phone calls, SMS) to use the application, what triggered them, frequency etc. It may be necessary to distinguish between the level of prompts/reminders required for the trial, and the level of prompts/reminders for a routine application outside of a RCT setting (discuss under item 21 – generalizability).

	1	2	3	4	5	
subitem not at all important	○	○	○	○	○	essential

Does your paper address subitem 5-xi? *

Copy and paste relevant sections from the manuscript (include quotes in quotation marks "like this" to indicate direct quotes from your manuscript), or elaborate on this item by providing additional information not in the ms, or briefly explain why the item is not applicable/relevant for your study

"In the event of non-completion of a session they also sent reminders."

These reminders were sent 7 days after the respective session should have been submitted. A maximum of three reminders (after 7, 14 and 21 days) were sent per session.

5-xii) Describe any co-interventions (incl. training/support)

Describe any co-interventions (incl. training/support): Clearly state any interventions that are provided in addition to the targeted eHealth intervention, as ehealth intervention may not be designed as stand-alone intervention. This includes training sessions and support [1]. It may be necessary to distinguish between the level of training required for the trial, and the level of training for a routine application outside of a RCT setting (discuss under item 21 – generalizability.

	1	2	3	4	5	
subitem not at all important	○	○	○	○	○	essential

Does your paper address subitem 5-xii? *

Copy and paste relevant sections from the manuscript (include quotes in quotation marks "like this" to indicate direct quotes from your manuscript), or elaborate on this item by providing additional information not in the ms, or briefly explain why the item is not applicable/relevant for your study

"Both groups had full access to treatment as usual."

6a) Completely defined pre-specified primary and secondary outcome measures, including how and when they were assessed

Does your paper address CONSORT subitem 6a? *

Copy and paste relevant sections from the manuscript (include quotes in quotation marks "like this" to indicate direct quotes from your manuscript), or elaborate on this item by providing additional information not in the ms, or briefly explain why the item is not applicable/relevant for your study

"All questionnaires were self-assessed online at baseline (T1), seven weeks (T2, post-treatment), 6 months (T3) and 12 months (T4, iSMI group only) after randomisation. The WLC group received access to the intervention following T3.

Primary outcome measure
The primary outcome was the level of perceived stress as measured by the PSS-10 [citation]. As this scale is based on Lazarus' transactional model of stress, it fits well with the theoretical basis of the intervention. We further decided to employ a general stress scale as previous research in a similar intervention for employees showed that work-related and non-work-related problems are equally often indicated and addressed [citation]. The items were answered using a 5-point Likert scale (0=never; 1=almost never; 2=sometimes; 3=fairly often; 4=very often; range 0-40) referring to the past week. Cronbach's alphas for this scale have been reported to range from .78 to .91 [citation] and was .90 at T2 in the present study.

Secondary outcome measures
Mental health: Among the secondary outcomes concerning mental health, the following outcomes were measured using the specified scales: depression (using the Center for Epidemiological Studies' Depression Scale, CES-D, [citation]; 20 items; range 0-60; α=.91), insomnia severity (using the Insomnia Severity Index, ISI, [39]; 7 items; range 0-28; α=.90), anxiety (using the subscale of the Hospital Anxiety and Depression Scales, HADS-A, [citation]; 7 items; range 0-21; α=.83), worrying (using the Penn State Worry Questionnaire, Ultra Brief Version-past week, PSWQ-PW, [citation]; 3 items; range 0-18; α=. 87) and quality of life (using the Short Form 12, SF-12, [citation]).
Work-related health: Within the area of work-related health we assessed emotional exhaustion (using the subscale of the Maslach Burnout Inventory, MBI-EE, [citation]; 5 items; range 1-6; α=.89), work engagement (using the Utrecht Work Engagement Scale, UWES, [citation]; 9 items; range 0-6; α=.94), and psychological detachment (using the subscale of the Recovery Experience Questionnaire,

REQ-PD, [citation]; 4 items; range 1-5; α=.93). Moreover, mean days of absenteeism and presenteeism within the previous three months were assessed using the respective items of the German Version of the Trimbos and Institute of Medical Technology Assessment Cost Questionnaire for Psychiatry (TiC–P-G; [citation]).
Skills/Competencies: Emotion regulation in terms of comprehension, acceptance, and self-support of the Emotion Regulation Skills Questionnaire (using the ERSQ-27-C, -A,-SS; [citation]; 9 items; range 0-4; α=.87, .86, .85), and regarding general distress (using the Emotion Specific Version, ERSQ-ES-GD, [citation]; 12 items; range 0-4; α=.88) were assessed as measures of skills/competencies.

Other measures
Client satisfaction (using the Client Satisfaction Questionnaire, CSQ-8, [citation]), demographic variables, and reasons for dropout will also be reported. "

6a-i) Online questionnaires: describe if they were validated for online use and apply CHERRIES items to describe how the questionnaires were designed/deployed

If outcomes were obtained through online questionnaires, describe if they were validated for online use and apply CHERRIES items to describe how the questionnaires were designed/deployed [9].

	1	2	3	4	5	
subitem not at all important	○	○	○	○	○	essential

Does your paper address subitem 6a-i?

Copy and paste relevant sections from manuscript text

6a-ii) Describe whether and how "use" (including intensity of use/dosage) was defined/measured/monitored

Describe whether and how "use" (including intensity of use/dosage) was defined/measured/monitored (logins, logfile analysis, etc.). Use/adoption metrics are important process outcomes that should be reported in any ehealth trial.

	1	2	3	4	5	
subitem not at all important	○	○	○	○	○	essential

Does your paper address subitem 6a-ii?

Copy and paste relevant sections from manuscript text

The number of completed sessions was used as a measure of adherence. The results are reported in the article.

6a-iii) Describe whether, how, and when qualitative feedback from participants was obtained

Describe whether, how, and when qualitative feedback from participants was obtained (e.g., through emails, feedback forms, interviews, focus groups).

1 2 3 4 5

subitem not at all important ○ ○ ○ ○ ○ essential

Does your paper address subitem 6a-iii?

Copy and paste relevant sections from manuscript text

Client satisfaction was obtained at post-test and is reported in the results section.

6b) Any changes to trial outcomes after the trial commenced, with reasons

Does your paper address CONSORT subitem 6b? *

Copy and paste relevant sections from the manuscript (include quotes in quotation marks "like this" to indicate direct quotes from your manuscript), or elaborate on this item by providing additional information not in the ms, or briefly explain why the item is not applicable/relevant for your study

There were no changes to trial outcomes.

7a) How sample size was determined

NPT: When applicable, details of whether and how the clustering by care provides or centers was addressed

7a-i) Describe whether and how expected attrition was taken into account when calculating the sample size

Describe whether and how expected attrition was taken into account when calculating the sample size.

	1	2	3	4	5	
subitem not at all important	○	○	○	○	○	essential

Does your paper address subitem 7a-i?

Copy and paste relevant sections from manuscript title (include quotes in quotation marks "like this" to indicate direct quotes from your manuscript), or elaborate on this item by providing additional information not in the ms, or briefly explain why the item is not applicable/relevant for your study

"Based on previous meta-analytic evidence on traditional stress management interventions [citation], an effect size of d=0.35 was expected. Therefore, based on an alpha of .05 (two-tailed test), and a power of 80%, a sample size of 132 participants per group was necessary."

7b) When applicable, explanation of any interim analyses and stopping guidelines

Does your paper address CONSORT subitem 7b? *

Copy and paste relevant sections from the manuscript (include quotes in quotation marks "like this" to indicate direct quotes from your manuscript), or elaborate on this item by providing additional information not in the ms, or briefly explain why the item is not applicable/relevant for your study

Not applicable

8a) Method used to generate the random allocation sequence

NPT: When applicable, how care providers were allocated to each trial group

Does your paper address CONSORT subitem 8a? *

Copy and paste relevant sections from the manuscript (include quotes in quotation marks "like this" to indicate direct quotes from your manuscript), or elaborate on this item by providing additional information not in the ms, or briefly explain why the item is not applicable/relevant for your study

"The applied random integer list was created by an independent researcher using a web-based randomisation program (randomisation.eu)."

8b) Type of randomisation; details of any restriction (such as blocking and block size)

Does your paper address CONSORT subitem 8b? *

Copy and paste relevant sections from the manuscript (include quotes in quotation marks "like this" to indicate direct quotes from your manuscript), or elaborate on this item by providing additional information not in the ms, or briefly explain why the item is not applicable/relevant for your study

"Using a 2-arm randomised controlled design, 264 participants were randomly allocated (at a ratio of 1:1 and a block size of 2) to the iSMI or to a WLC group."

9) Mechanism used to implement the random allocation sequence (such as sequentially numbered containers), describing any steps taken to conceal the sequence until interventions were assigned

Does your paper address CONSORT subitem 9? *

Copy and paste relevant sections from the manuscript (include quotes in quotation marks "like this" to indicate direct quotes from your manuscript), or elaborate on this item by providing additional information not in the ms, or briefly explain why the item is not applicable/relevant for your study

"Using a 2-arm randomised controlled design, 264 participants were randomly allocated (at a ratio of 1:1 and a block size of 2) to the iSMI or to a WLC group. "

"The applied random integer list was created by an independent researcher using a web-based randomisation program (randomisation.eu). The participants were informed about the randomisation outcome via e-mail."

10) Who generated the random allocation sequence, who enrolled participants, and who assigned participants to interventions

Does your paper address CONSORT subitem 10? *

Copy and paste relevant sections from the manuscript (include quotes in quotation marks "like this" to indicate direct quotes from your manuscript), or elaborate on this item by providing additional information not in the ms, or briefly explain why the item is not applicable/relevant for your study

"The applied random integer list was created by an independent researcher using a web-based randomisation program (randomisation.eu). The participants were informed about the randomisation outcome via e-mail."

11a) If done, who was blinded after assignment to interventions (for example, participants, care providers, those assessing outcomes) and how

NPT: Whether or not administering co-interventions were blinded to group assignment

11a-i) Specify who was blinded, and who wasn't

Specify who was blinded, and who wasn't. Usually, in web-based trials it is not possible to blind the participants [1, 3] (this should be clearly acknowledged), but it may be possible to blind outcome assessors, those doing data analysis or those administering co-interventions (if any).

	1	2	3	4	5	
subitem not at all important	○	○	○	○	○	essential

Does your paper address subitem 11a-i? *

Copy and paste relevant sections from the manuscript (include quotes in quotation marks "like this" to indicate direct quotes from your manuscript), or elaborate on this item by providing additional information not in the ms, or briefly explain why the item is not applicable/relevant for your study

Researchers, e-coaches and participants were not blinded (not possible for this type of trial).

" The participants were informed about the randomisation outcome via e-mail."

11a-ii) Discuss e.g., whether participants knew which intervention was the "intervention of interest" and which one was the "comparator"

Informed consent procedures (4a-ii) can create biases and certain expectations - discuss e.g., whether participants knew which intervention was the "intervention of interest" and which one was the "comparator".

	1	2	3	4	5	
subitem not at all important	○	○	○	○	○	essential

Does your paper address subitem 11a-ii?

Copy and paste relevant sections from the manuscript (include quotes in quotation marks "like this" to

indicate direct quotes from your manuscript), or elaborate on this item by providing additional information not in the ms, or briefly explain why the item is not applicable/relevant for your study

"The participants were informed about the randomisation outcome via e-mail."

11b) If relevant, description of the similarity of interventions

(this item is usually not relevant for ehealth trials as it refers to similarity of a placebo or sham intervention to a active medication/intervention)

Does your paper address CONSORT subitem 11b? *

Copy and paste relevant sections from the manuscript (include quotes in quotation marks "like this" to indicate direct quotes from your manuscript), or elaborate on this item by providing additional information not in the ms, or briefly explain why the item is not applicable/relevant for your study

Not applicable

12a) Statistical methods used to compare groups for primary and secondary outcomes

NPT: When applicable, details of whether and how the clustering by care providers or centers was addressed

Does your paper address CONSORT subitem 12a? *

Copy and paste relevant sections from the manuscript (include quotes in quotation marks "like this" to indicate direct quotes from your manuscript), or elaborate on this item by providing additional information not in the ms, or briefly explain why the item is not applicable/relevant for your study

"All analyses are reported according to the Consolidated Standards of Reporting Trials (CONSORT) statement using intention-to-treat procedures (ITT). Additionally, per-protocol and study completers-only analyses are reported. A significance level of .05 (two-sided) was used for all analyses. Analyses were performed using IBM SPSS version 22. "

"The iSMI and WLC groups were compared at 7 weeks (T2) and 6 months (T3) using ANCOVA with baseline levels as covariates. Cohen's d with 95% confidence intervals (CIs) was calculated based on the imputed dataset by comparing the means and SDs of the iSMI and WLC groups at the respective time points (e.g., post-means and post-SDs). According to Cohen [citation], d=0.2 can be considered a small effect, d=0.5 a medium effect and d=0.8 a large effect. "

12a-i) Imputation techniques to deal with attrition / missing values

Imputation techniques to deal with attrition / missing values: Not all participants will use the intervention/comparator as intended and attrition is typically high in ehealth trials. Specify how participants who did not use the application or dropped out from the trial were treated in the statistical analysis (a complete case analysis is strongly discouraged, and simple imputation techniques such as LOCF may also be problematic [4]).

	1	2	3	4	5	
subitem not at all important	○	○	○	○	○	essential

Does your paper address subitem 12a-i? *

Copy and paste relevant sections from the manuscript (include quotes in quotation marks "like this" to indicate direct quotes from your manuscript), or elaborate on this item by providing additional information not in the ms, or briefly explain why the item is not applicable/relevant for your study

"Multiple imputation was used to handle missing data [citation]. Ten single imputations of the missing values were calculated based on the valid data for all outcome measures at all assessment points (T1, T2, T3, and T4) as well as age and gender and were aggregated into a single overall estimate of the effects of the intervention. "

12b) Methods for additional analyses, such as subgroup analyses and adjusted analyses

Does your paper address CONSORT subitem 12b? *

Copy and paste relevant sections from the manuscript (include quotes in quotation marks "like this" to indicate direct quotes from your manuscript), or elaborate on this item by providing additional information not in the ms, or briefly explain why the item is not applicable/relevant for your study

"Reliable change: The clinical significance in terms of reliable change was calculated according to the method of Jacobson and Truax [citation]. The participants were defined as having reliably changed if their PSS-10 score differed more than (+/-) 5.16 points from T1-T2 and T1-T3.
Symptom-free status: According to Jacobson and Truax [citation], a cut-off point indicating symptom-free status was calculated and defined as scoring more than 2 SDs below the mean (T1) of the stressed population.
Number needed to treat: The number needed to treat (NNT) indicates the number of participants who must be treated to generate one additional clinically significant change. NNTs and their 95% confidence intervals [citation] were calculated for reliable change and symptom-free status."

X26) REB/IRB Approval and Ethical Considerations [recommended as subheading under "Methods"] (not a CONSORT item)

X26-i) Comment on ethics committee approval

	1	2	3	4	5	
subitem not at all important	○	○	○	○	○	essential

Does your paper address subitem X26-i?

Copy and paste relevant sections from the manuscript (include quotes in quotation marks "like this" to indicate direct quotes from your manuscript), or elaborate on this item by providing additional information not in the ms, or briefly explain why the item is not applicable/relevant for your study

"All procedures involved in the study were consistent with the generally accepted standards of ethical practice and were approved by the ethical committee of the University of Marburg (reference number AZ 2012-43K)."

x26-ii) Outline informed consent procedures

Outline informed consent procedures e.g., if consent was obtained offline or online (how? Checkbox, etc.?), and what information was provided (see 4a-ii). See [6] for some items to be included in informed consent documents.

	1	2	3	4	5	
subitem not at all important	○	○	○	○	○	essential

Does your paper address subitem X26-ii?

Copy and paste relevant sections from the manuscript (include quotes in quotation marks "like this" to indicate direct quotes from your manuscript), or elaborate on this item by providing additional information not in the ms, or briefly explain why the item is not applicable/relevant for your study

An e-mail including a PDF of the informed consent was sent to the applicants. The applicants were asked to print the informed consent, sign it and send it via post or scanned via e-mail.

"[...] applicants had to submit their signed informed consent via post or scanned via e-mail [...]"

X26-iii) Safety and security procedures

Safety and security procedures, incl. privacy considerations, and any steps taken to reduce the likelihood or detection of harm (e.g., education and training, availability of a hotline)

	1	2	3	4	5	
subitem not at all important	○	○	○	○	○	essential

Does your paper address subitem X26-iii?

Copy and paste relevant sections from the manuscript (include quotes in quotation marks "like this" to indicate direct quotes from your manuscript), or elaborate on this item by providing additional information not in the ms, or briefly explain why the item is not applicable/relevant for your study

"Those interested in participating had to provide an e-mail address and a first and last name which could be pseudonyms if desired. "

"Any applicants who were at risk of suicide (Beck Suicide Item > 1; [citation]) or previously diagnosed with dissociative or psychotic symptoms were excluded."

RESULTS

13a) For each group, the numbers of participants who were randomly assigned, received intended treatment, and were analysed for the primary outcome

NPT: The number of care providers or centers performing the intervention in each group and the number of patients treated by each care provider in each center

Does your paper address CONSORT subitem 13a? *

Copy and paste relevant sections from the manuscript (include quotes in quotation marks "like this" to indicate direct quotes from your manuscript), or elaborate on this item by providing additional information not in the ms, or briefly explain why the item is not applicable/relevant for your study

Please refer to CONSORT Flow diagram. An intention-to-treat analysis was conducted.

Dropout attrition: "Overall, 21 (8.0%) participants at T2, 28 (10.6%) participants at T3, and 40 (30.3%, iSMI only) participants at T4 did not provide follow-up data for the primary outcome. A somewhat higher dropout rate was observed for the iSMI group (T2: n=16, T3: n=17) compared with the WLC (T2: n=5, T3: n=11). Thereby, groups significantly differed at T2 (Chi2=6.26; P<.05), but not at T3. Participants who did not provide follow-up data did not differ in a meaningful way from those who provided data, neither on baseline stress scores or any other baseline outcomes, with the exception of worrying (P<.05)."

"Nonusage attrition: Of the 132 individuals participating in the iSMI, session 1 was completed by 122 of the participants (92.4%), session 2 by 117 (88.6%), session 3 by 112 (84.8%), session 4 by 108 (81.8%), session 5 by 103 (78.0%), session 6 by 97 (73.5 %), session 7 by 93 (70.5%), and the booster session by 72 (54.5%) of the participants. Because of a lack of time and changes in personal circumstances, 10 participants (7.6%) did not start the iSMI. Nine participants (6.8%) reported reasons for discontinuing the iSMI; these included lack of time (n=4), lack of motivation (n=3), technical problems (n=1), and dissatisfaction with the intervention (n=1). On average, the participants in the iSMI group completed 5.70 (SD=2.32) out of the 7 modules (81.4% of the intervention) and used the iSMI for 8.27 weeks (SD=8.54; range 0-56)."

13b) For each group, losses and exclusions after randomisation, together with reasons

Does your paper address CONSORT subitem 13b? (NOTE: Preferably, this is shown in a CONSORT flow diagram) *

Copy and paste relevant sections from the manuscript (include quotes in quotation marks "like this" to indicate direct quotes from your manuscript), or elaborate on this item by providing additional information not in the ms, or briefly explain why the item is not applicable/relevant for your study

Please refer to CONSORT Flow diagram.

13b-i) Attrition diagram

Strongly recommended: An attrition diagram (e.g., proportion of participants still logging in or using the intervention/comparator in each group plotted over time, similar to a survival curve) or other figures or tables demonstrating usage/dose/engagement.

	1	2	3	4	5	
subitem not at all important	○	○	○	○	○	essential

Does your paper address subitem 13b-i?

Copy and paste relevant sections from the manuscript or cite the figure number if applicable (include quotes in quotation marks "like this" to indicate direct quotes from your manuscript), or elaborate on this item by providing additional information not in the ms, or briefly explain why the item is not applicable/relevant for your study

"Nonusage attrition: Of the 132 individuals participating in the iSMI, session 1 was completed by 122 of the participants (92.4%), session 2 by 117 (88.6%), session 3 by 112 (84.8%), session 4 by 108 (81.8%), session 5 by 103 (78.0%), session 6 by 97 (73.5 %), session 7 by 93 (70.5%), and the booster session by 72 (54.5%) of the participants. Because of a lack of time and changes in personal circumstances, 10 participants (7.6%) did not start the iSMI. Nine participants (6.8%) reported reasons for discontinuing the iSMI; these included lack of time (n=4), lack of motivation (n=3), technical problems (n=1), and dissatisfaction with the intervention (n=1). On average, the participants in the iSMI group completed 5.70 (SD=2.32) out of the 7 modules (81.4% of the intervention) and used the iSMI for 8.27 weeks (SD=8.54; range 0-56)."

14a) Dates defining the periods of recruitment and follow-up

Does your paper address CONSORT subitem 14a? *

Copy and paste relevant sections from the manuscript (include quotes in quotation marks "like this" to indicate direct quotes from your manuscript), or elaborate on this item by providing additional information not in the ms, or briefly explain why the item is not applicable/relevant for your study

"Recruitment occurred from January to October 2013"

"All questionnaires were self-assessed online at baseline (T1), seven weeks (T2, post-treatment), 6 months (T3) and 12 months (T4, iSMI group only) after randomisation."

14a-i) Indicate if critical "secular events" fell into the study period

Indicate if critical "secular events" fell into the study period, e.g., significant changes in Internet resources available or "changes in computer hardware or Internet delivery resources"

	1	2	3	4	5	
subitem not at all important	○	○	○	○	○	essential

Does your paper address subitem 14a-i?

Copy and paste relevant sections from the manuscript (include quotes in quotation marks "like this" to indicate direct quotes from your manuscript), or elaborate on this item by providing additional information not in the ms, or briefly explain why the item is not applicable/relevant for your study

No secular events occurred.

14b) Why the trial ended or was stopped (early)

Does your paper address CONSORT subitem 14b? *

Copy and paste relevant sections from the manuscript (include quotes in quotation marks "like this" to indicate direct quotes from your manuscript), or elaborate on this item by providing additional information not in the ms, or briefly explain why the item is not applicable/relevant for your study

The trial was completed as planned.

15) A table showing baseline demographic and clinical characteristics for each group

NPT: When applicable, a description of care providers (case volume, qualification, expertise, etc.) and centers (volume) in each group

Does your paper address CONSORT subitem 15? *

Copy and paste relevant sections from the manuscript (include quotes in quotation marks "like this" to indicate direct quotes from your manuscript), or elaborate on this item by providing additional information not in the ms, or briefly explain why the item is not applicable/relevant for your study

Please refer to Table 1 (baseline characteristics) and Table 2 (means and standard deviations for each group).

15-i) Report demographics associated with digital divide issues

In ehealth trials it is particularly important to report demographics associated with digital divide issues, such as age, education, gender, social-economic status, computer/Internet/ehealth literacy of the participants, if known.

	1	2	3	4	5	
subitem not at all important	○	○	○	○	○	essential

Does your paper address subitem 15-i? *

Copy and paste relevant sections from the manuscript (include quotes in quotation marks "like this" to indicate direct quotes from your manuscript), or elaborate on this item by providing additional information not in the ms, or briefly explain why the item is not applicable/relevant for your study

Please refer to Table 1 (baseline characteristics).

16) For each group, number of participants (denominator) included in each analysis and whether the analysis was by original assigned groups

16-i) Report multiple "denominators" and provide definitions

Report multiple "denominators" and provide definitions: Report N's (and effect sizes) "across a range of study participation [and use] thresholds" [1], e.g., N exposed, N consented, N used more than x times, N used more than y weeks, N participants "used" the intervention/comparator at specific pre-defined time points of interest (in absolute and relative numbers per group). Always clearly define "use" of the intervention.

	1	2	3	4	5	
subitem not at all important	○	○	○	○	○	essential

Does your paper address subitem 16-i? *

Copy and paste relevant sections from the manuscript (include quotes in quotation marks "like this" to indicate direct quotes from your manuscript), or elaborate on this item by providing additional information not in the ms, or briefly explain why the item is not applicable/relevant for your study

Please refer to the results section.

16-ii) Primary analysis should be intent-to-treat

Primary analysis should be intent-to-treat, secondary analyses could include comparing only "users", with the appropriate caveats that this is no longer a randomized sample (see 18-i).

	1	2	3	4	5	
subitem not at all important	○	○	○	○	○	essential

Does your paper address subitem 16-ii?

Copy and paste relevant sections from the manuscript (include quotes in quotation marks "like this" to indicate direct quotes from your manuscript), or elaborate on this item by providing additional information not in the ms, or briefly explain why the item is not applicable/relevant for your study

Intention-to-treat analyses have been performed.

17a) For each primary and secondary outcome, results for each group, and the estimated effect size and its precision (such as 95% confidence interval)

Does your paper address CONSORT subitem 17a? *

Copy and paste relevant sections from the manuscript (include quotes in quotation marks "like this" to indicate direct quotes from your manuscript), or elaborate on this item by providing additional information not in the ms, or briefly explain why the item is not applicable/relevant for your study

The estimated between-group effect sizes and their 95% confidence intervals are reported in Table 3.

17a-i) Presentation of process outcomes such as metrics of use and intensity of use

In addition to primary/secondary (clinical) outcomes, the presentation of process outcomes such as metrics of use and intensity of use (dose, exposure) and their operational definitions is critical. This does not only refer to metrics of attrition (13-b) (often a binary variable), but also to more continuous exposure metrics such as "average session length". These must be accompanied by a technical description how a metric like a "session" is defined (e.g., timeout after idle time) [1] (report under item 6a).

	1	2	3	4	5	
subitem not at all important	◯	◯	◯	◯	◯	essential

Does your paper address subitem 17a-i?

Copy and paste relevant sections from the manuscript (include quotes in quotation marks "like this" to indicate direct quotes from your manuscript), or elaborate on this item by providing additional information not in the ms, or briefly explain why the item is not applicable/relevant for your study

"Of the 132 individuals participating in the iSMI, session 1 was completed by 122 of the participants (92.4%), session 2 by 117 (88.6%), session 3 by 112 (84.8%), session 4 by 108 (81.8%), session 5 by 103 (78.0%), session 6 by 97 (73.5 %), session 7 by 93 (70.5%), and the booster session by 72 (54.5%) of the participants. Because of a lack of time and changes in personal circumstances, 10 participants (7.6%) did not start the iSMI. Nine participants (6.8%) reported reasons for discontinuing the iSMI; these included lack of time (n=4), lack of motivation (n=3), technical problems (n=1), and dissatisfaction with the intervention (n=1). On average, the participants in the iSMI group completed 5.70 (SD=2.32) out of the 7 modules (81.4% of the intervention) and used the iSMI for 8.27 weeks (SD=8.54; range 0-56)."

17b) For binary outcomes, presentation of both absolute and relative effect sizes is recommended

Does your paper address CONSORT subitem 17b? *

Copy and paste relevant sections from the manuscript (include quotes in quotation marks "like this" to indicate direct quotes from your manuscript), or elaborate on this item by providing additional information not in the ms, or briefly explain why the item is not applicable/relevant for your study

"Reliable change: At T2, more participants in the iSMI group (n=81, 61.4%) showed reliable improvement on the PSS-10 compared with the WLC (n=33, 25.0%). A reliable deterioration was present in n=2 (1.5%) of the iSMI and n=11 (8.3%) of the WLC, whereas 49 (37.1%; iSMI) and 88 (66.7%; WLC) were reliably unchanged. At T3, those showing reliable improvement numbered n=102 (77.3%) in the iSMI and n=44 (33.3%) in the WLC group. Those showing reliable deterioration numbered n=1 in the iSMI (0.8%) and n=8 (6.1%) in the WLC group. No reliable change was present in n=29 (22.0%; iSMI) and n=80 (60.6%; WLC). The NNTs for reliable improvement were 2.75 (95% CI: 2.11-3.96) at T2 and 2.28 (95% CI: 1.83-3.01) at T3. The groups significantly differed from T1-T2 (Chi2=37.54, P<.001) and from T1-T3 (Fisher's exact=53.53, P<.001).
Symptom-free status: In this study, the cut-off score was 17.70 and below indicating a value of 2 SDs below the mean of the stressed population at T1 (M=25.52, SD=3.91). More participants in the iSMI group met the criterion for full remission of stress symptoms

compared with the WLC group at T2 (iSMI: n=68, 51.5%; WLC: n=26, 19.7%; Chi2=29.14, P<.001; NNT=3.14, 95% CI: 2.34-4.78) and T3 (iSMI: n=79, 59.8%; WLC: n=31, 23.5%; Chi2=35.91, P<.001; NNT=2.75, 95% CI: 2.11-3.95)."

18) Results of any other analyses performed, including subgroup analyses and adjusted analyses, distinguishing pre-specified from exploratory

Does your paper address CONSORT subitem 18? *

Copy and paste relevant sections from the manuscript (include quotes in quotation marks "like this" to indicate direct quotes from your manuscript), or elaborate on this item by providing additional information not in the ms, or briefly explain why the item is not applicable/relevant for your study

"Intervention completion: A separate per-protocol analysis was conducted for participants who completed the intervention (≥6 sessions) which was defined as working through all of the theoretical intervention content which was presented up to session 6. The ANCOVA showed significant differences between the subsample of intervention completers (n=97) and the WLC (n=132) with regard to perceived stress in favour of the experimental condition at T2 (F(1,226)=66.85, P<.001) and T3 (F(1,226)=74.70, P<.001) with slightly higher effect sizes at T2 (d=0.95; CI: 0.69-1.20) and T3 (d=1.05; CI: 0.79-1.31) as compared to the total iSMI sample. Within the iSMI group, we further compared intervention completers to non-completers. The ANCOVA showed a significant difference for reduction of perceived stress at T2 (F(1,129)=7.76, P=.006), but not at T3 or T4.

Text message support: There were no significant differences in the primary outcome between participants who received text messages and those who did not, nor was there any significant difference depending on the level of intensity of the individually chosen text message support."

18-i) Subgroup analysis of comparing only users

A subgroup analysis of comparing only users is not uncommon in ehealth trials, but if done, it must be stressed that this is a self-selected sample and no longer an unbiased sample from a randomized trial (see 16-iii).

	1	2	3	4	5	
subitem not at all important	○	○	○	○	○	essential

Does your paper address subitem 18-i?

Copy and paste relevant sections from the manuscript (include quotes in quotation marks "like this" to indicate direct quotes from your manuscript), or elaborate on this item by providing additional information not in the ms, or briefly explain why the item is not applicable/relevant for your study

"Intervention completion: A separate per-protocol analysis was conducted for participants who completed the intervention (≥6 sessions) which was defined as working through all of the theoretical intervention content which was presented up to session 6. The ANCOVA showed significant differences between the subsample of intervention completers (n=97) and the WLC (n=132) with regard to perceived stress in favour of the experimental condition at T2 (F(1,226)=66.85, P<.001) and T3 (F(1,226)=74.70, P<.001) with slightly higher effect sizes at T2 (d=0.95; CI: 0.69-1.20) and T3 (d=1.05; CI: 0.79-1.31) as compared to the total iSMI sample. Within the iSMI group, we further compared intervention completers to non-completers. The ANCOVA showed a significant difference for reduction of perceived stress at T2 (F(1,129)=7.76, P=.006), but not at T3 or T4."

19) All important harms or unintended effects in each group

(for specific guidance see CONSORT for harms)

Does your paper address CONSORT subitem 19? *

Copy and paste relevant sections from the manuscript (include quotes in quotation marks "like this" to indicate direct quotes from your manuscript), or elaborate on this item by providing additional information not in the ms, or briefly explain why the item is not applicable/relevant for your study

Not applicable

19-i) Include privacy breaches, technical problems

Include privacy breaches, technical problems. This does not only include physical "harm" to participants, but also incidents such as perceived or real privacy breaches [1], technical problems, and other unexpected/unintended incidents. "Unintended effects" also includes unintended positive effects [2].

	1	2	3	4	5	
subitem not at all important	◯	◯	◯	◯	◯	essential

Does your paper address subitem 19-i?

Copy and paste relevant sections from the manuscript (include quotes in quotation marks "like this" to indicate direct quotes from your manuscript), or elaborate on this item by providing additional information not in the ms, or briefly explain why the item is not applicable/relevant for your study

No privacy breaches, major technical problems or unexpected/unintended incidents occurred.

19-ii) Include qualitative feedback from participants or observations from staff/researchers

Include qualitative feedback from participants or observations from staff/researchers, if available, on strengths and shortcomings of the application, especially if they point to unintended/unexpected effects or uses. This includes (if available) reasons for why people did or did not use the application as intended by the developers.

1 2 3 4 5

Does your paper address subitem 19-ii?

Copy and paste relevant sections from the manuscript (include quotes in quotation marks "like this" to indicate direct quotes from your manuscript), or elaborate on this item by providing additional information not in the ms, or briefly explain why the item is not applicable/relevant for your study

"Compared with the available intervention completion rates (e.g., 38.5%, [citation]; 44.0%, [citation]), the percentage of participants adhering completing the intervention was high in this study (70.5%). Considerable efforts were undertaken to increase adherence through methods that are generally considered to be effective, including human support [citation], interactive exercises [citation], tailoring of the intervention [citation] and reminders [citation] via mobile phone."

DISCUSSION

22) Interpretation consistent with results, balancing benefits and harms, and considering other relevant evidence

NPT: In addition, take into account the choice of the comparator, lack of or partial blinding, and unequal expertise of care providers or centers in each group

22-i) Restate study questions and summarize the answers suggested by the data, starting with primary outcomes and process outcomes (use)

Restate study questions and summarize the answers suggested by the data, starting with primary outcomes and process outcomes (use).

	1	2	3	4	5	
subitem not at all important	○	○	○	○	○	essential

Does your paper address subitem 22-i? *

Copy and paste relevant sections from the manuscript (include quotes in quotation marks "like this" to indicate direct quotes from your manuscript), or elaborate on this item by providing additional information not in the ms, or briefly explain why the item is not applicable/relevant for your study

"The primary aim of the present study was to evaluate the efficacy of a guided iSMI for employees. For this purpose, a two-arm, waitlist-control randomised trial was conducted. The results indicate that the training is highly effective in reducing employees' stress level in the short (d=0.83) and longer term (d=1.02) compared with the levels observed in a waitlist control group. Reduced stress levels in the iSMI group could be maintained up to 12 months. Significant medium to large between-group effects were also found for relevant secondary outcomes concerning mental health (e.g., depression), work-related health (e.g., emotional exhaustion), and stress-related skills (e.g., emotion regulation competencies)."

22-ii) Highlight unanswered new questions, suggest future research

Highlight unanswered new questions, suggest future research.

	1	2	3	4	5	
subitem not at all important	○	○	○	○	○	essential

Does your paper address subitem 22-ii?

Copy and paste relevant sections from the manuscript (include quotes in quotation marks "like this" to indicate direct quotes from your manuscript), or elaborate on this item by providing additional information not in the ms, or briefly explain why the item is not applicable/relevant for your study

"Future research should replicate the results of this trial and investigate the moderators of outcome and adherence. It is also of interest whether the coaching time spent on each individual (up to 4 hours) could be reduced without losses in treatment effects, thereby resulting in more economical versions of iSMI. Moreover, future research should test iSMIs against the gold standard in the field (i.e., face-to-face interventions) and assess which training format works best for which type of participant and under what circumstances. Although both formats may be equally effective, they may work differently on participants with varying personal characteristics and web-based interventions may be more advantageous in terms of efficiency and costs."

20) Trial limitations, addressing sources of potential bias, imprecision, and, if relevant, multiplicity of analyses

20-i) Typical limitations in ehealth trials

Typical limitations in ehealth trials: Participants in ehealth trials are rarely blinded. Ehealth trials often look at a multiplicity of outcomes, increasing risk for a Type I error. Discuss biases due to non-use of the intervention/usability issues, biases through informed consent procedures, unexpected events.

	1	2	3	4	5	
subitem not at all important	○	○	○	○	○	essential

Does your paper address subitem 20-i? *

Copy and paste relevant sections from the manuscript (include quotes in quotation marks "like this" to indicate direct quotes from your manuscript), or elaborate on this item by providing additional information not in the ms, or briefly explain why the item is not applicable/relevant for your study

"The following limitations of this trial must be acknowledged. First, for feasibility reasons, only self-report measures were assessed. Although the replacement of self-report measures with physiological measures is not recommended in occupational stress research [citation], a combination of both could produce further valuable insights. Second, because this study was in the setting of indicated prevention, these results only account for participants showing relatively high baseline scores. Thus, no conclusions can be drawn regarding participants with lower stress levels (e.g., in a universal prevention setting). Third, with regard to the generalisability of results, the facts that the participants self-selected into the trial, the majority

were female, and individuals working in the social sector were slightly overrepresented need to be taken into consideration. Fourth, to determine the added value of the mobile component providing "in-the-moment" support and encouragement, direct comparison studies would be needed comparing the intervention with and without mobile support. Finally, it is important to acknowledge that some improvements were also observed in the WLC group over time; in fact, this pattern has previously been found in other trials [citation]."

21) Generalisability (external validity, applicability) of the trial findings

NPT: External validity of the trial findings according to the intervention, comparators, patients, and care providers or centers involved in the trial

21-i) Generalizability to other populations

Generalizability to other populations: In particular, discuss generalizability to a general Internet population, outside of a RCT setting, and general patient population, including applicability of the study results for other organizations

	1	2	3	4	5	
subitem not at all important	○	○	○	○	○	essential

Does your paper address subitem 21-i?

Copy and paste relevant sections from the manuscript (include quotes in quotation marks "like this" to indicate direct quotes from your manuscript), or elaborate on this item by providing additional information not in the ms, or briefly explain why the item is not applicable/relevant for your study

"[...] with regard to the generalisability of results, the facts that the participants self-selected into the trial, the majority were female, and individuals working in the social sector were slightly overrepresented need to be taken into consideration."

21-ii) Discuss if there were elements in the RCT that would be different in a routine application setting

Discuss if there were elements in the RCT that would be different in a routine application setting (e.g., prompts/reminders, more human involvement, training sessions or other co-interventions) and what impact the omission of these elements could have on use, adoption, or outcomes if the intervention is applied outside of a RCT setting.

	1	2	3	4	5	
subitem not at all important	○	○	○	○	○	essential

Does your paper address subitem 21-ii?

Copy and paste relevant sections from the manuscript (include quotes in quotation marks "like this" to indicate direct quotes from your manuscript), or elaborate on this item by providing additional information not in the ms, or briefly explain why the item is not applicable/relevant for your study

Due to the elaborated study inclusion process, the sample was presumably highly motivated to change their behaviour.

"It is also of interest whether the coaching time spent on each individual (up to 4 hours) could be reduced without losses in treatment effects, thereby resulting in more economical versions of iSMI. "

" [...] to determine the added value of the mobile component providing "in-the-moment" support and encouragement, direct comparison studies would be needed comparing the intervention with and without mobile support."

OTHER INFORMATION

23) Registration number and name of trial registry

Does your paper address CONSORT subitem 23? *

Copy and paste relevant sections from the manuscript (include quotes in quotation marks "like this" to indicate direct quotes from your manuscript), or elaborate on this item by providing additional information not in the ms, or briefly explain why the item is not applicable/relevant for your study

Registration: German Register Clinical Trials, DRKS00004749: http://drks-neu.uniklinik-freiburg.de/drks_web/setLocale_EN.do

24) Where the full trial protocol can be accessed, if available

Does your paper address CONSORT subitem 24? *

Cite a Multimedia Appendix, other reference, or copy and paste relevant sections from the manuscript (include quotes in quotation marks "like this" to indicate direct quotes from your manuscript), or elaborate on this item by providing additional information not in the ms, or briefly explain why the item is not applicable/relevant for your study

Heber, E., Ebert, D., Lehr, D., Nobis, S., Berking, M., Riper, H. (2013). Efficacy and cost-effectiveness of a web-based and mobile stress-management intervention for employees: design of a randomized controlled trial. BMC Public Health 13:655.

http://www.biomedcentral.com/1471-2458/13/655

25) Sources of funding and other support (such as supply of drugs), role of funders

Does your paper address CONSORT subitem 25? *

Copy and paste relevant sections from the manuscript (include quotes in quotation marks "like this" to indicate direct quotes from your manuscript), or elaborate on this item by providing additional information not in the ms, or briefly explain why the item is not applicable/relevant for your study

"We would like to acknowledge the European Union (EFRE) for funding this project within the Lueneburg Innovation Incubator, TM 1.1 (CCI 2007DE161PR001). [...] This study was partially funded by the health insurance company "Barmer GEK."

X27) Conflicts of Interest (not a CONSORT item)

X27-i) State the relation of the study team towards the system being evaluated

In addition to the usual declaration of interests (financial or otherwise), also state the relation of the study team towards the system being evaluated, i.e., state if the authors/evaluators are distinct from or identical with the developers/sponsors of the intervention.

	1	2	3	4	5	
subitem not at all important	○	○	○	○	○	essential

Does your paper address subitem X27-i?

Copy and paste relevant sections from the manuscript (include quotes in quotation marks "like this" to indicate direct quotes from your manuscript), or elaborate on this item by providing additional information not in the ms, or briefly explain why the item is not applicable/relevant for your study

"Dr. Dirk Lehr, Dr. David Ebert, and Prof. Matthias Berking are stakeholders of the "Institute for Online Health Trainings", that aims to transfer scientific knowledge related to the presented research into routine health care. The Institute was founded in January 2015. Since August 2015, Elena Heber is an employee at this Institute. At the time of planning, conducting, and evaluating the study, the Institute did not yet exist."

About the CONSORT EHEALTH checklist

As a result of using this checklist, did you make changes in your manuscript? *

- ○ yes, major changes
- ◉ yes, minor changes
- ○ no

What were the most important changes you made as a result of using this checklist?

How much time did you spend on going through the checklist INCLUDING making changes in your manuscript *

3

As a result of using this checklist, do you think your manuscript has improved? *

- (x) yes
- ( ) no
- ( ) Sonstiges:

Would you like to become involved in the CONSORT EHEALTH group?

This would involve for example becoming involved in participating in a workshop and writing an "Explanation and Elaboration" document

- ( ) yes
- (x) no
- ( ) Sonstiges:

Any other comments or questions on CONSORT EHEALTH

STOP - Save this form as PDF before you click submit

To generate a record that you filled in this form, we recommend to generate a PDF of this page (on a Mac, simply select "print" and then select "print as PDF") before you submit it.

When you submit your (revised) paper to JMIR, please upload the PDF as supplementary file.

Don't worry if some text in the textboxes is cut off, as we still have the complete information in our database. Thank you!

Final step: Click submit !

Click submit so we have your answers in our database!

Senden

Geben Sie niemals Passwörter über Google Formulare weiter.

Bereitgestellt von Google Forms

Dieser Inhalt wurde nicht von Google erstellt und wird von Google auch nicht unterstützt.

Missbrauch melden - Nutzungsbedingungen - Zusätzliche Bestimmungen
